# How Microscale Flow and Vapor Transport Shape New Particle Formation and Growth

**DOI:** 10.1021/acs.est.6c10206

**Published:** 2026-08-17

**Authors:** Kunal Ghosh, Gargi Sengupta, Suneeti Mishra

**Affiliations:** † School of Earth, Environment and Sustainability, 4468 University of Leeds, Leeds LS2 9JT, U.K.; ‡ Environmental Research Group, School of Public Health, Imperial College London, White City Campus, Wood Lane, London W12 0BZ, U.K.; § 28498 PSI Center for Energy and Environmental Sciences, Villigen 5232, Switzerland

**Keywords:** New particle formation, aerosol microphysics, 3D modeling, nanoparticle growth, Particle growth rate, vapor transport, high resolution, 3D-ICAM

## Abstract

Accurate simulation of atmospheric new particle formation (NPF) remains limited because large-scale models cannot resolve aerosol processes at fine spatial scales, while detailed microphysics models assume spatially homogeneous conditions. Here we use a high-resolution three-dimensional multiphysics framework (3D-ICAM) to quantify how environmental heterogeneity governs NPF dynamics at meter scales. Simulations at 10 m resolution across contrasting emission geometries reveal that spatial gradients in wind and precursor vapor convergence substantially modify local nucleation intensity, particle growth rates, and size-distribution structure. Under weak-wind conditions (<1 m s^–1^) the model reproduces nucleation “hotspots”, intermittent multiepisode nucleation, and persistently bimodal size distributions absent under homogeneous conditions. Comparison with an equivalent box (0D) model isolates the effect of spatial structure: resolving it raises predicted growth rates at the survival-limiting sub-3 nm sizes by up to ∼20× and brings modeled growth within the range of field observations, whereas the box model underpredicts across the size spectrum. This occurs because localized, transport-generated microenvironments relax the growth–scavenging competition that governs particle survival. Meter-scale environmental structure therefore strongly influences urban NPF and must be considered when interpreting observations, constraining nucleation mechanisms, and predicting particle number and cloud condensation nuclei budgets.

## Graphical abstract

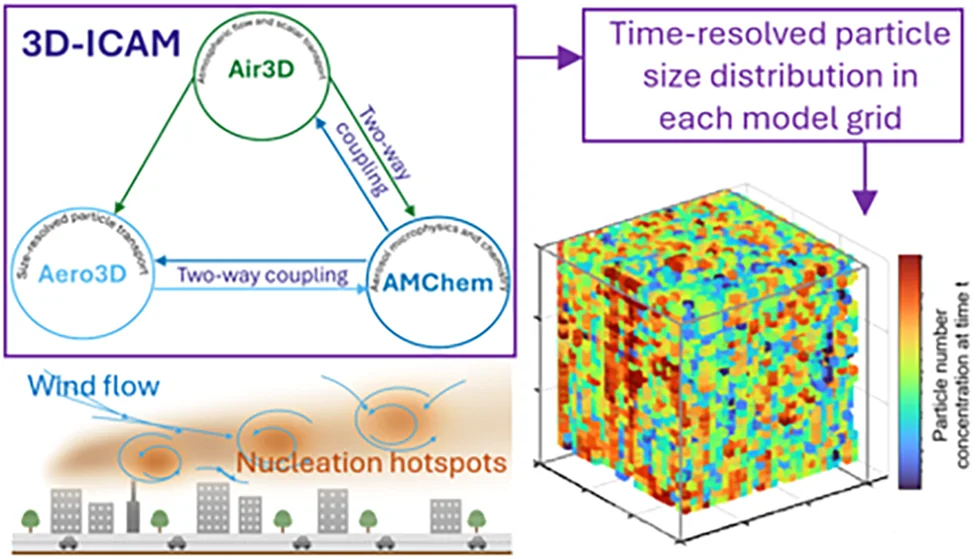

## Introduction

New particle formation (NPF) is a major source of atmospheric aerosol particles and plays a critical role in shaping cloud properties, radiative forcing, and air quality.^1^ It refers to the formation of nanometer-scale molecular clusters that grow into particles capable of acting as cloud condensation nuclei (CCN).^2^ NPF contributes a substantial fraction of global CCN, reaching up to 80% in some regions,^3^ and influences both background particle populations and pollution episodes.^4^ In urban settings, freshly formed particles interact with local emissions and boundary-layer dynamics, whereas in remote or marine environments NPF is frequently the dominant particle source. Although chemical pathways involved in nucleation are increasingly well understood, the environmental controls that modulate how these pathways manifest in real atmospheric settings remain incompletely characterized, particularly in heterogeneous urban landscapes.^5^ Understanding the interplay between chemistry, meteorology, and emissions is therefore essential for improving air quality forecasts and reducing uncertainties in aerosol–climate interactions.

Over the past two decades, advances in observational techniques have greatly improved the mechanistic understanding of NPF. Field campaigns employing condensation particle counters (CPCs), scanning mobility particle sizers (SMPS), and chemical ionization mass spectrometry (CIMS) have characterized precursor vapors such as sulfuric acid, ammonia, and amines with molecular-level detail.^6^ Long-term measurements at ground-based networks, including the SMEAR stations in Finland, have quantified NPF frequency, seasonality, and growth rates across diverse environments.^5,7–11^ Machine learning and image-recognition approaches have improved consistency in event classification,^5,12,13^ while high-resolution mass spectrometry has revealed the composition of early clusters, including acid–base complexes and highly oxygenated organic molecules.^3^ These advances have provided unprecedented chemical and temporal detail, but they also underscore the need for models that can interpret observations within environmentally realistic, spatially structured settings.

Despite extensive observational progress, current modeling frameworks struggle to resolve the spatial and temporal complexity of NPF in real environments. Global climate models (GCMs) operate at coarse spatial resolutions and represent aerosols with a small number of size modes.^14,15^ Regional climate and large-eddy simulation models achieve finer spatial resolution but still rely on simplified aerosol microphysics that cannot represent early stage cluster formation. These frameworks are valuable for assessing large-scale aerosol–climate interactions, yet they are not designed to capture microscale variability relevant for NPF in polluted or topographically complex regions.^3^ In contrast, zero-dimensional (0D) box models^4,16^ and one-dimensional parcel models^17,18^ resolve detailed chemistry and microphysics using tens to hundreds of size bins, but they assume spatial homogeneity and therefore cannot represent flow-driven vapor accumulation, dilution, or horizontal gradients. This mismatch between highly resolved observational data sets and spatially coarse or homogeneous modeling frameworks limits our ability to interpret NPF dynamics in real-world conditions.

To address this gap, we apply a high-resolution three-dimensional multiphysics modeling tool (3D-ICAM) to investigate how environmental heterogeneity modulates NPF. 3D-ICAM resolves particle dynamics at 10 m resolution and couples size-resolved aerosol microphysics with evolving meteorological fields through two-way feedbacks. The framework integrates a computational fluid dynamics module for wind, temperature, humidity, and emissions; an aerosol chemistry and microphysics module that simulates nucleation, condensation, coagulation, and deposition; and a particle transport module that advects and diffuses size-resolved particle distributions. This structure enables mechanistic investigation of how flow structures and environmental gradients shape NPF behavior. These are features absent from both coarse-resolution climate models and nonspatial box models. To the best of our knowledge, this is the first study to examine NPF at meter-scale (∼10 m) resolution in a fully coupled 3D domain, enabling direct exploration of how environmental processes amplify, suppress, or modify chemically driven nucleation and growth.

## Methodology

We apply a Three-Dimensional Integrated Chemistry–Aerosol–Microphysics model (3D-ICAM) to simulate size-resolved new particle formation (NPF) with full spatial and temporal resolution in an urban-scale domain. The model resolves coupled fluid dynamics, gas–aerosol chemistry, and particle microphysics to capture how evolving environmental structure influences nucleation and early growth.

Simulations are conducted in a 10^9^ m^3^ domain (1 km × 1 km × 1 km) discretized at 10 m resolution. 3D-ICAM outputs three-dimensional fields of particle number concentration (d*N*/d log *D*_p_), nucleation-mode particle abundance (*D*_p_ ≤ 3 nm), and size-dependent growth rates throughout the domain.

### Model Structure

3D-ICAM consists of three dynamically coupled components (Figure 1). The **Air3D** module solves the atmospheric flow, temperature, humidity, and precursor vapor fields using a computational fluid dynamics (CFD) framework. The **AMChem** module simulates gas-phase chemistry and aerosol microphysics, including cluster formation, nucleation, condensation, coagulation, and deposition across 80 size bins. The **Aero3D** module transports aerosol populations by advection and turbulent diffusion.

**Figure 1:**
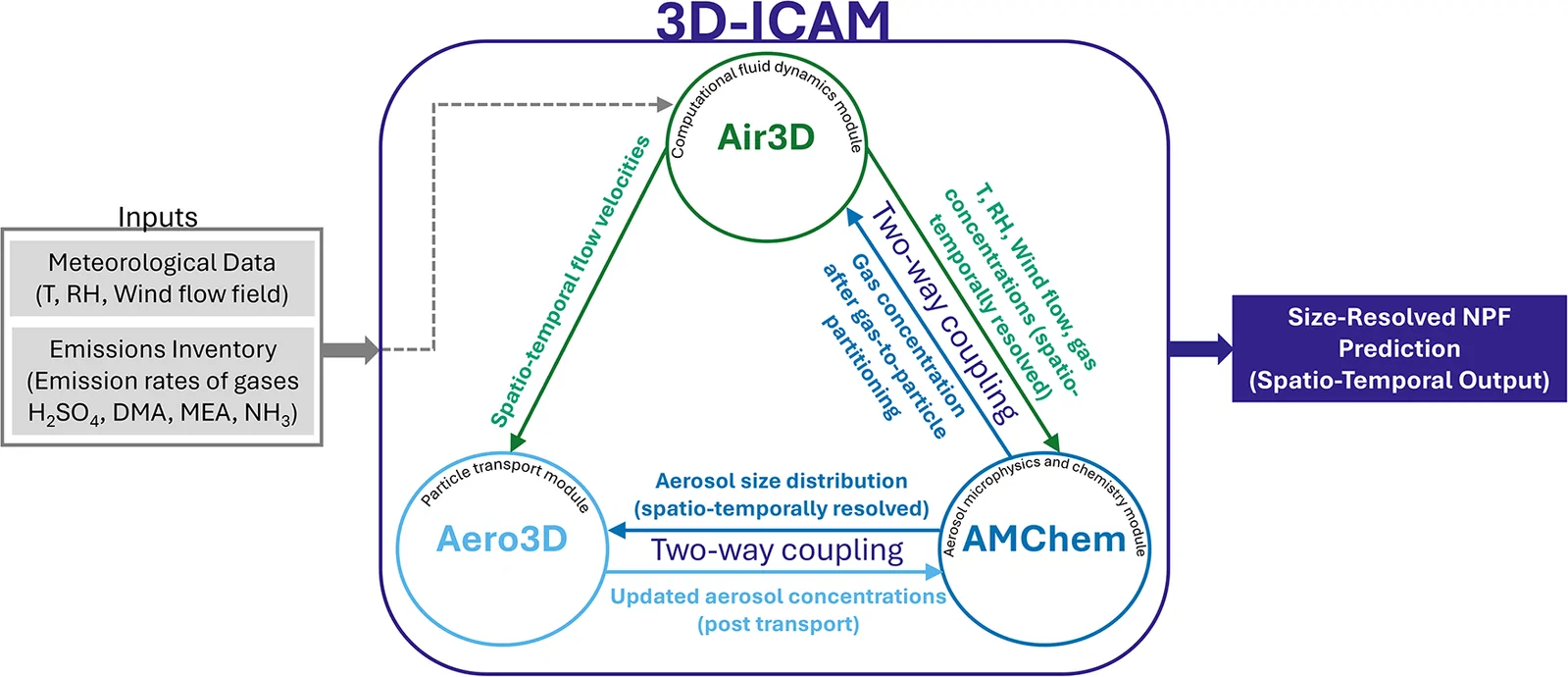
Schematic of the 3D-ICAM model architecture, showing the coupling between Air3D (flow and meteorology), AMChem (aerosol chemistry and microphysics), and Aero3D (size-resolved transport) modules. The model uses meteorological and emissions data as input and simulates size-resolved new particle formation (NPF) with full spatial and temporal resolution.

At each time step, Air3D provides updated meteorological and vapor fields to AMChem, which computes nucleation and growth rates. Aero3D then transports the size-resolved aerosols, and resulting concentrations feed back to Air3D and AMChem. This fully coupled loop allows changes in flow, dilution, and vapor convergence to directly influence particle formation and vice versa.

#### Air3D: Atmospheric Flow and Scalar Transport

Air3D solves the conservation equations for mass, momentum, energy, and scalar species using a finite-volume approach within a Reynolds-averaged Navier–Stokes (RANS) framework with a standard *k*–ε turbulence closure. The model represents three-dimensional advection and turbulent mixing of precursor vapors, which determine local saturation ratios and thereby regulate nucleation and condensational growth. In the present configuration, temperature and relative humidity are assumed to be spatially uniform, and buoyancy-driven convection is not explicitly represented. The complete governing equations, numerical discretization details, and turbulence parametrizations are provided in the Supporting Information (Section S1) and follow the formulation of Ghosh et al.^19^

The model operates at a grid spacing of 10 m and resolves spatial variability in the mean flow and scalar fields across the domain. Turbulent transport is represented through effective eddy viscosity and diffusivity, such that turbulent motions are treated statistically rather than explicitly resolved. This formulation captures spatial gradients and heterogeneity in vapor concentrations, which govern local saturation conditions and thus control the rates of nucleation and condensational growth. The resolved flow field therefore provides the physical structure through which transport and chemical processes are coupled.

#### AMChem: Aerosol Microphysics and Chemistry

The AMChem module simulates aerosol dynamics using a sectional representation of particle sizes. It is built on the general dynamic equation framework of Ghosh et al.^19^ and includes nucleation, coagulation, condensation, and deposition processes.

Nucleation is represented using a cluster-kinetic scheme based on the four-acid–four-base (*A*_4_ *B*_4_) formulation of Brean et al.,^4^ which describes the formation of thermodynamically stable clusters from sulfuric acid (H_2_SO_4_) and a generic base (B) such as dimethylamine (DMA), monoethanolamine (MEA), or NH_3_. The parametrization accounts for acid–base collision rates, cluster evaporation, and losses of vapors to the pre-existing condensation sink. The full expression for the nucleation rate and all associated parameters are given in the Supporting Information (Section S2).

Coagulation is treated by combining five physical kernels representing different collision pathways: Brownian motion, convective Brownian diffusion enhancement, gravitational collection, turbulent inertial motion, and turbulent shear.

The kernels include slip-flow corrections and, where relevant, van der Waals interactions, following Vohra et al.^20^ and Ghosh et al.^19^ Condensational growth is driven by H_2_SO_4_ vapor and is computed using a modified expression based on Laakso et al.,^21^ neglecting charge effects but retaining size dependence and transition-regime corrections. Size-resolved dry deposition to the ground is implemented using size-dependent deposition velocities that account for gravitational settling and near-surface turbulent transport, following Ghosh et al.,^19^ Vohra et al.^20^

All microphysical processes are evaluated in 80 logarithmically spaced size bins spanning 1–5000 nm. AMChem is advanced in time using a semi-implicit numerical scheme designed to conserve aerosol mass and number and to maintain numerical stability at high particle number concentrations.^22^ Full mathematical formulations and numerical details are provided in the Supporting Information (Section S2).

#### Aero3D: Size-Resolved Particle Transport

The Aero3D module simulates the three-dimensional transport and dispersion of aerosols across the domain. It evolves the number concentration of particles in each size bin as a function of space and time, driven by advection and turbulent diffusion using the flow and turbulence fields provided by Air3D.

Effective particle diffusivities combine Brownian diffusion (computed via the Stokes–Einstein relation for each size bin) and turbulent eddy diffusion derived from the local turbulence characteristics. This formulation allows Aero3D to represent size-dependent spreading of freshly formed particles and the development of spatial gradients in nucleation-mode and larger particles. The underlying transport equation, numerical solution method, and treatment of coupling with AMChem are described in the Supporting Information (Section S3), following the approach of Ghosh et al.^19^

### Model Setup and Numerical Configuration

The 3D-ICAM simulations are performed in a cubic computational domain of volume 10^9^ m^3^ (1000 m × 1000 m × 1000 m), discretized using a uniform 10 m grid in all three spatial directions (Figure 2). Each simulation is run for 24 h with a fixed time step of 1 min.

**Figure 2:**
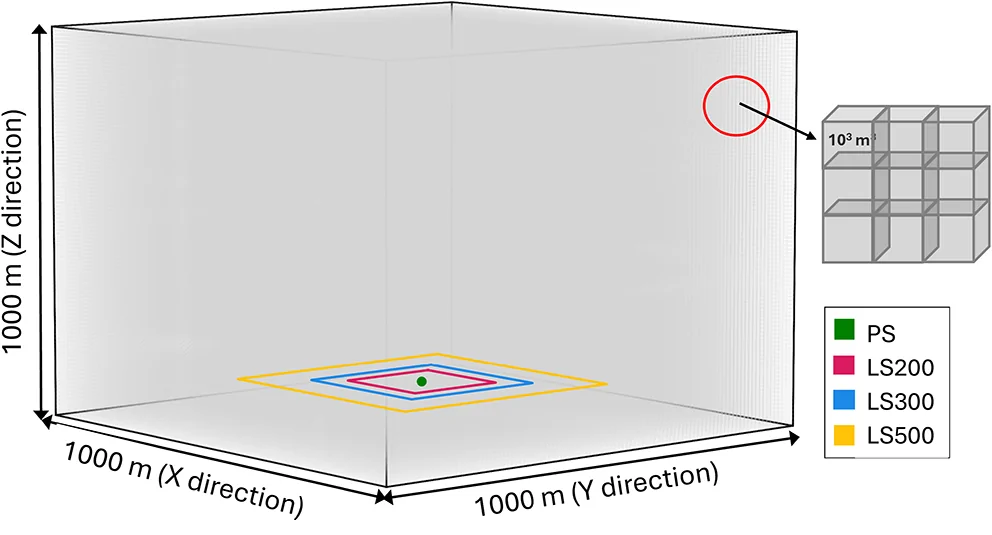
Computational domain used in the 3D-ICAM simulations. The domain is a cube of volume 10^9^ m^3^ (1000 m × 1000 m × 1000 m), discretized using a uniform structured grid with 10^3^ m^3^ resolution. Emission configurations include a point source (PS, green) at the center of the domain at 30 m height and three line sources (LS200, pink; LS300, blue; LS500, yellow) forming square perimeters at 30 m height with side lengths of 200, 300, and 500 m, respectively.

To evaluate how the spatial configuration of emissions influences NPF, we consider four emission geometries: one point source (PS) and three line sources (LS200, LS300, LS500). The point source is located at the center of the horizontal domain, 30 m above ground level. The three line sources form square perimeters at 30 m height, centered at the domain midpoint, with side lengths of 200, 300, and 500 m, respectively. The 30 m height is chosen to represent typical rooftop-level or stack emissions in an urban neighborhood.^23^ These configurations allow us to isolate the effect of emission extent and geometry on nucleation intensity, growth rates, and spatial patterns of particle number concentrations under otherwise identical meteorological drivers.

Aerosol particle sizes are represented using 80 logarithmically spaced bins from 1 to 5000 nm. Key initial and boundary conditions are summarized in Table 1, with additional details provided in the Supporting Information (Section S4).

**Table 1:** Summary of Initial and Boundary Conditions for the 3D-ICAM Simulations

Parameter	Value/treatment
Domain size	1000 m × 1000 m × 1000 m
Grid resolution	10 m (uniform)
Total number of grid cells	10^6^
Simulation duration	24 h
Time step	1 min
Numerical integration	Implicit (Air3D); semi-implicit (AMChem, Aero3D)
Temperature	5–25 °C, time-varying, spatially uniform^6^
Horizontal wind field	Hourly values from Mishra et al. 2023,^6^ nudged each hour
Vertical velocity	Initial vertical velocity 0.1 m s^–1^ (used for flow initialization only)
Relative humidity	35–90% over 24 h^6^
Emitted species	H_2_SO_4_, DMA, MEA, NH_3_
H_2_SO_4_ precursor forcing	Time-varying diurnal H_2_SO_4_ profile with daytime maxima of order 10^7^ molecules cm^–3^, based on Cai et al. 2021.^24^ Applied as the time-dependent source strength Φ_H_2_SO_4__(*t*) within the prescribed source region.
Base precursor forcing	DMA, MEA, and NH_3_ reference source strengths of 10^7^ molecules cm^–3^ h, following Brean et al. 2023.
Emission-geometry normalization	For all precursor source terms, including H_2_SO_4_ and bases, the per-cell source strength is scaled by Ω_ref_/Ω_src_, so that the total precursor input integrated over the source region is identical across PS, LS200, LS300, and LS500.
Amine chemical loss	Photochemical amine loss is not explicitly represented, to remain consistent with the 0-D reference framework.
Initial aerosol distribution	Log-normal, mode 170 nm, 20–1000 nm, total 10^5^ m^–3^;^6^ applied uniformly across all grid cells.
Aerosol size representation	80 logarithmically spaced bins (1–5000 nm)
Hygroscopicity (κ)	0 (nonhygroscopic)
Particle density	1.83 g cm^–3^
Nucleation scheme	*J*_A4B4_ for H_2_SO_4_–base clusters^4^

The model is implemented in MATLAB R2023b and parallelized using GPU acceleration (NVIDIA GeForce RTX 3060 Ti) to make meter-scale simulations over 10^6^ grid cells computationally feasible. The semi-implicit treatment of aerosol microphysics and the implicit flow solver together enable numerically stable, fully coupled 24 h simulations for each emission scenario. Additional numerical performance details are provided in the Supporting Information (Section S4).

## Results and Discussion

### Wind Flow Field

Figure 3 shows the three-dimensional wind velocity field simulated by the Air3D module at four time points over the 24 h simulation: 6 h (panel a), 12 h (panel b), 18 h (panel c), and 24 h (panel d). The color shading represents wind speed (in m s^–1^), while the overlaid arrows indicate the local flow direction at each grid point.

**Figure 3:**
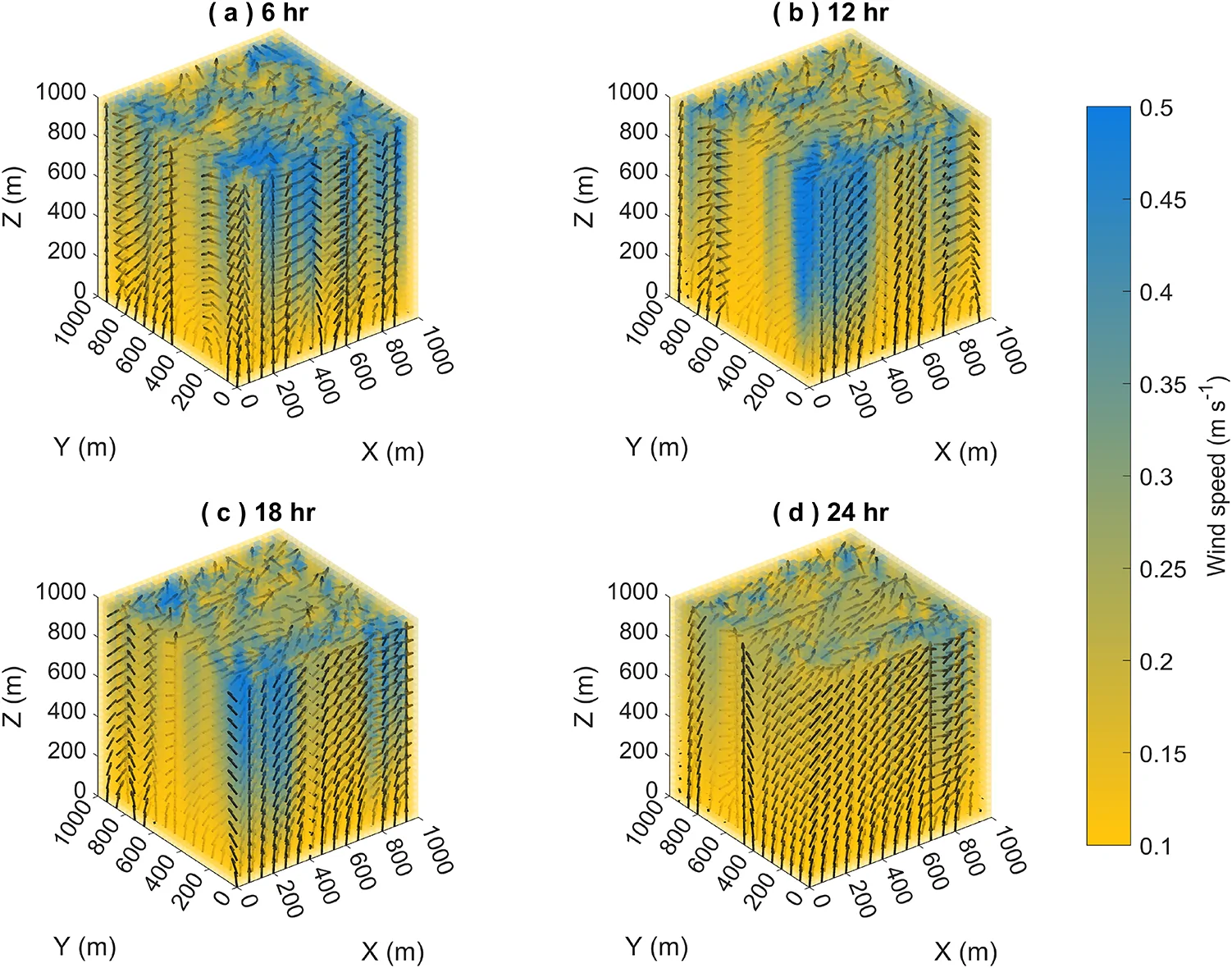
Three-dimensional wind velocity field simulated by the Air3D module at four time points during the 24-h simulation: (a) 6 h, (b) 12 h, (c) 18 h, and (d) 24 h. Wind speed (in m s^–1^) is represented by the color scale, while black arrows indicate the direction of the velocity vectors at each cubic grid point.

Under weak large-scale forcing (wind speeds < 0.5 m s^–1^), the simulated flow develops substantial internal variability over time. Even at early stages, the velocity field shows spatially varying directions near the surface, indicating low-level shear and localized recirculation. As the simulation progresses, regions of differing wind speed emerge across the domain, particularly at intermediate times (e.g., 12 and 18 h), reflecting increasing vertical and horizontal structure in the flow.

By 24 h, localized zones with misaligned flow vectors and enhanced mixing become more pronounced, consistent with the emergence of turbulence-like structures. These features arise from mechanically driven processes within the flow, including advection and shear-induced mixing represented through the turbulence closure. As a result, under weak-wind conditions, the model produces a dynamically evolving velocity field in which spatial variability emerges at meter-scale resolution.

To assess the robustness of this spatial variability, the dependence of turbulent kinetic energy (TKE, m^2^ s^–2^) and the turbulence length scale (*L*_*t*_ = *k* ^3/2^/*ε*, m) on grid spacing (Δ*x*) is analyzed and shown in SI Figures S6.6 and S6.7. At coarse resolutions (Δ*x* ≳ 300–500 m), the simulated flow is weak and characterized by unrealistically large length scales (e.g., *L*_*t*_ > 2000 m), indicating that the dominant flow structures are not resolved. Such values are far larger than expected turbulence scales in weak-wind urban environments, where energy-containing eddies are typically of order 10–100 m^25^ and are constrained by surface roughness, shear, and local forcing.

This overestimation of the turbulence length scale arises from under-resolved velocity gradients, which lead to artificially low dissipation rates (ε). With grid refinement, gradients and shear are better resolved, increasing dissipation and reducing *L*_*t*_ toward physically meaningful values. Below approximately 30–20 m resolution, both TKE and *L*_*t*_ converge, with only minor differences between the 10 and 5 m simulations. This convergence indicates that the dominant flow–transport structures are adequately captured at a resolution of 10 m under the weak-wind conditions considered here, such that further refinement does not significantly change the simulated flow. This supports the interpretation that the spatial variability in Figure 3 reflects physically driven flow dynamics rather than numerical artifacts.

### Aerosol Number Concentrations

Figure 4 shows the spatio-temporal evolution of the number concentration of nucleation-mode particles of 1–3 nm (*N*_1–3_) over the 24 h simulation period for the four emission geometries. Across all configurations, *N*_1–3_ rapidly becomes distributed throughout the domain, indicating that nucleation and transport occur concurrently, with newly formed particles quickly advected away from their source regions. At all times, the particle field exhibits strong spatial heterogeneity, with high-concentration regions (≳ 10^10^m^–3^) interspersed with areas several orders of magnitude lower, forming complex three-dimensional patterns.

**Figure 4:**
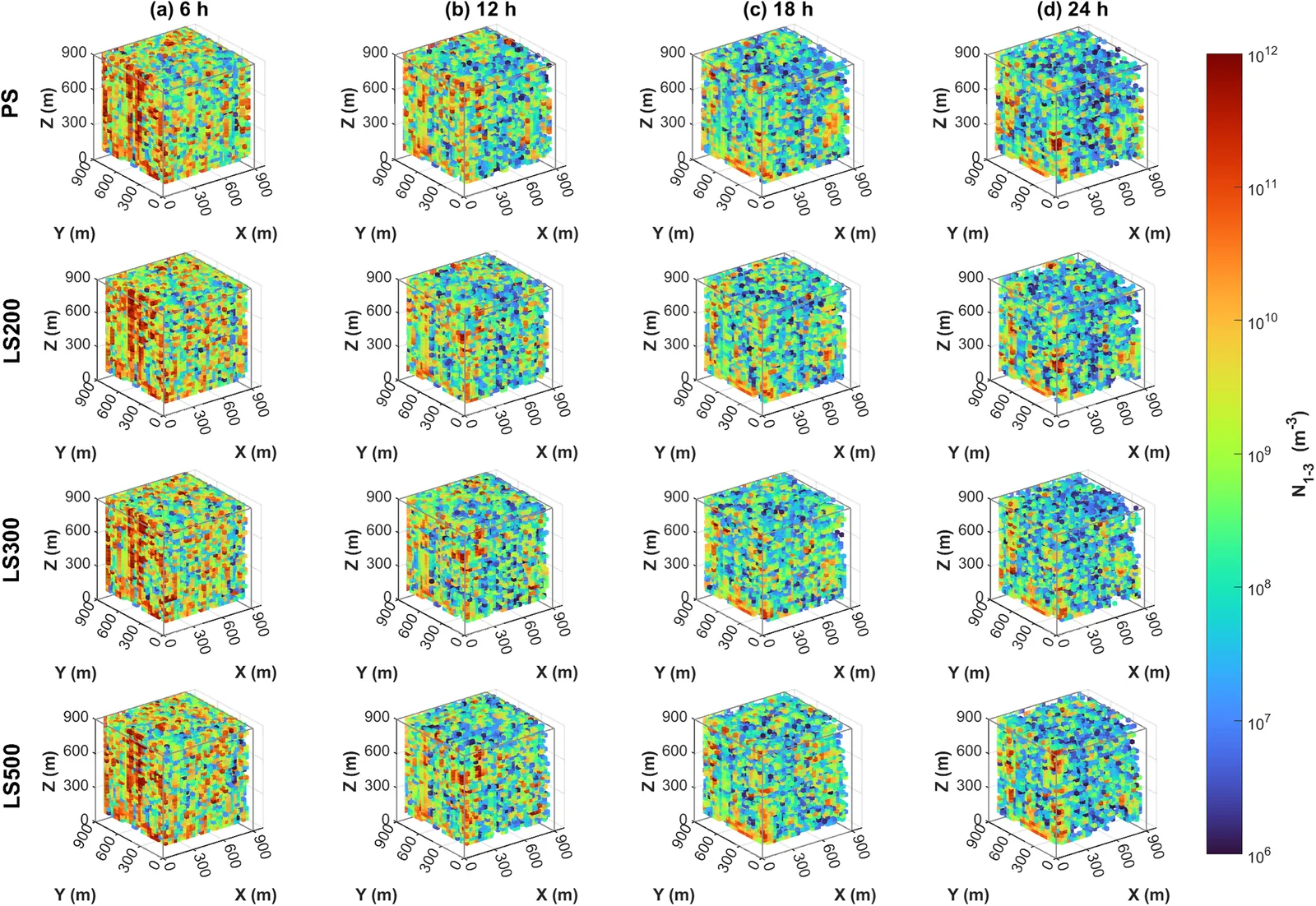
Spatio-temporal evolution of the total number concentration of nucleation-mode particles (1–3 nm), denoted by *N*_1–3_ (in m^–3^), at four time points from the start of simulation: (a) 6 h, (b) 12 h, (c) 18 h, and (d) 24 h. Each row corresponds to a different initial emission geometry case: (i) point source (PS), (ii) line source with 200 m side length (LS200), (iii) line source with 300 m side length (LS300), and (iv) line source with 500 m side length (LS500). Colors indicate *N*_1–3_ on a logarithmic scale.

At 6 h (Figure 4a), the spatial heterogeneity is already well developed. The PS case shows vertically extended regions of elevated *N*_1–3_, whereas the LS configurations produce more laterally distributed but still highly nonuniform structures. Despite identical total precursor input, localized hotspots emerge in all cases, indicating that precursor availability alone does not determine where nucleation occurs.

As the simulation progresses (12–24 h), the spatial patterns evolve substantially. High-concentration regions become more fragmented and shift in location, while the overall magnitude of *N*_1–3_ decreases. This decline is consistent with particle growth beyond the 3 nm threshold, as supported by the sustained *N*_3–10_ concentrations in Figure S6.1. Differences between emission geometries are most pronounced at early times, with line sources producing broader regions of elevated *N*_1–3_ than the point source. However, these differences become less distinct at later times as atmospheric transport redistributes particles and vapors, reducing the direct imprint of the initial emission geometry. This partial homogenization reflects the controlled conditions of the present simulations, namely a single emission event and weak, slowly varying winds that limit large-scale ventilation. In real environments, where emissions are continuous and meteorology varies over multiple timescales, this homogenization is unlikely to persist, and ongoing emission–flow interactions should instead sustain heterogeneous particle distributions.

Comparison with the wind field (Figure 3) provides further insight into the origin of the spatial patterns. Regions of elevated *N*_1–3_ (red–orange) tend to occur in areas of relatively weak or variable flow, where reduced ventilation increases vapor residence time, allowing precursor accumulation. In contrast, regions with higher wind speeds (blue shading in Figure 3) generally correspond to lower *N*_1–3_, consistent with enhanced dilution and transport. The evolving wind structure, characterized by directional shear, stratification, and localized mixing, modulates the redistribution of both vapors and particles, causing the locations of high and low *N*_1–3_ to shift over time. This relationship is further supported by the spatial distributions of sulfuric acid concentration and nucleation rate (SI Figures S6.3 and S6.4), which show that regions of elevated *N*_1–3_ coincide with localized maxima in precursor concentration and nucleation activity.

Overall, these results demonstrate that nucleation-mode particle concentrations are inherently three-dimensional and dynamically structured. Even with identical total precursor input, the interaction between emission geometry and atmospheric transport governs the spatial organization of new particle formation.

### Growth Rate

Figure 5 shows the three-dimensional distribution of particle growth rates (GR) for four size ranges: 1–3, 3–10, 10–25, and 25–40 nm. Across all emission geometries, GR is strongly spatially heterogeneous, with localized regions of rapid growth interspersed with cells of much slower growth within the same size range. This heterogeneity mirrors the structure already seen in the wind field (Figure 3) and in the nucleation-mode number concentration (Figure 4): growth is not a domain-uniform process, even though total emissions, chemistry, and microphysical parametrizations are identical across the simulations.

**Figure 5:**
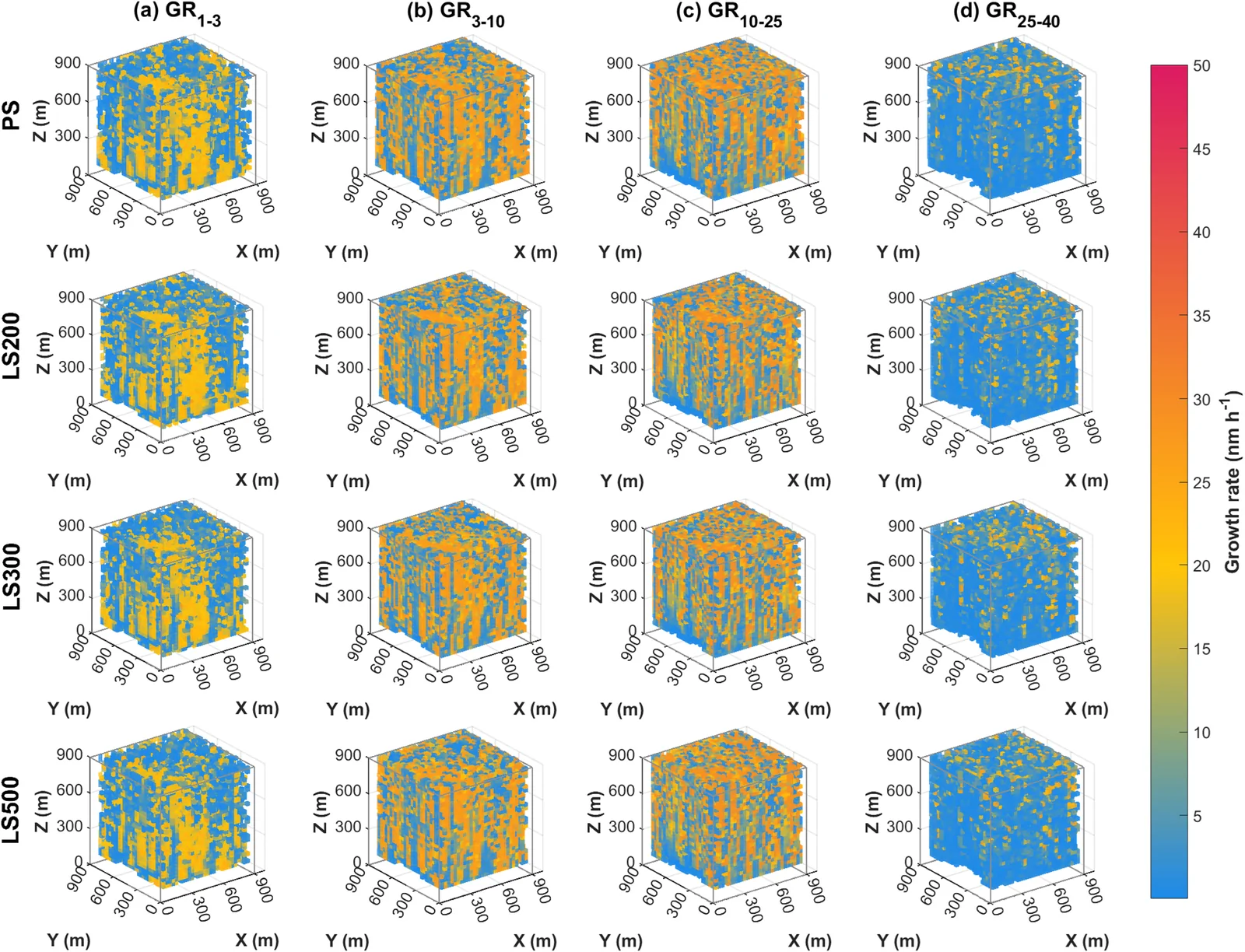
Three-dimensional distribution of particle growth rates (GR, nm h^–1^) for four size ranges: (a) 1–3 nm, (b) 3–10 nm, (c) 10–25 nm, and (d) 25–40 nm. Rows correspond to different emission geometries: point source (PS), and line sources with characteristic lengths of 200 m (LS200), 300 m (LS300), and 500 m (LS500). Colors indicate the magnitude of the growth rate.

The growth field exhibits a pronounced size dependence. Pooling all grid cells with a trackable growing mode, the median GR increases from ∼15 nm h^–1^ in the 1–3 nm range to a maximum of ∼25 nm h^–1^ in the 3–10 nm range, remains high at ∼19 nm h^–1^ in the 10–25 nm range, and then drops sharply to ∼1.6 nm h^–1^ in the 25–40 nm range (Figure 5a–d). The fraction of cells containing a reliably tracked growing mode follows a related trend, rising from ∼38% at 1–3 nm to ∼66% at 3–10 nm and ∼71% at 10–25 nm, before falling to ∼57% at 25–40 nm. Growth is thus most vigorous and most spatially widespread through the intermediate 3–25 nm range, where particles have passed the earliest, loss-dominated stage and condense efficiently while precursor vapor remains available.

The two ends of the size range are shaped by different limitations. At the smallest sizes (1–3 nm; Figure 5a), a trackable growing mode is present in only ∼ 38% of cells and the growth field is the most patchy of all four ranges. This is the most vulnerable stage of particle evolution, where condensational growth competes with the Kelvin barrier and with losses by coagulation, dilution, and transport away from vapor-rich regions, so that only a fraction of newly formed particles encounter conditions favorable for continued growth. The survival of particles through this bottleneck is therefore set by local microenvironmental conditions rather than by the domain-mean precursor abundance. At the largest sizes (25–40 nm; Figure 5d), growth slows markedly (median ∼1.6 nm h^–1^): once the population has grown through the smaller ranges, condensable vapor is depleted and the larger particles themselves provide an increasing sink, so further condensational growth becomes less significant. The size sequence in Figure 5a–d thus reflects a transition from a survival-limited regime at 1–3 nm, through a vapor-rich growth maximum at 3–25 nm, to a vapor-limited regime at 25–40 nm.

Differences between emission geometries are comparatively subtle. All four reproduce the same qualitative size dependence and the same strongly heterogeneous spatial texture, and the median GR varies by less than ∼1 nm h^–1^ across geometries in the 3–10, 10–25, and 25–40 nm ranges; only in the 1–3 nm range does the cross-geometry spread reach a few nm h^–1^. The response is not simply proportional to source extent: the largest line source (LS500) does not produce uniformly enhanced growth but remains spatially structured, consistent with stronger dilution and redistribution of vapors over its larger footprint. Emission geometry therefore influences where growth is favored, but the growth field is ultimately shaped by the coupled action of emissions and transport.

These patterns follow the evolving wind field (Figure 3), with enhanced growth in vapor convergence zones and slower growth where dilution reduces vapor exposure; the condensation-sink field (Figure S6.5) similarly sets where growing particles are scavenged or survive. Growth is thus coupled to the same flow-driven vapor fields as nucleation and *N*_1–3_ (Figure 4).

### Impact of 3D Environment in Simulations

To isolate the role of spatial dynamics in NPF, we compared the output of the full 3D-ICAM model to a 0D version of the simulation that includes only the chemistry and aerosol microphysics module (AMChem), without the CFD or particle transport components. This 0D configuration is equivalent to a box model and represents a spatially homogeneous environment subject only to time-varying precursor emissions and meteorological conditions. It is consistent in structure with other state-of-the-art 0D frameworks.^4,16^ For direct comparison, we extracted time series output from the central grid point of the 3D domain at 30 m height and compared it with the 0D model output under identical input conditions.

Figure 6 shows the evolution of particle number size distributions over a 24 h period at the central grid point (30 m height) for the 0D reference model (panel a) and the four 3D emission scenarios (panels b–e). Banana-shaped size distributions are a well-known feature of atmospheric NPF events, displaying a characteristic upward-curving trajectory in *dN*/*d* log *D*_*p*_ as particles form and grow from nanometer sizes. In the 0D model, which includes chemistry and microphysics but excludes transport and environmental structure, the banana shape is smooth, continuous, and symmetric, with growth beginning near midday and closely resembling the well-known type Ia event.^5^

**Figure 6:**
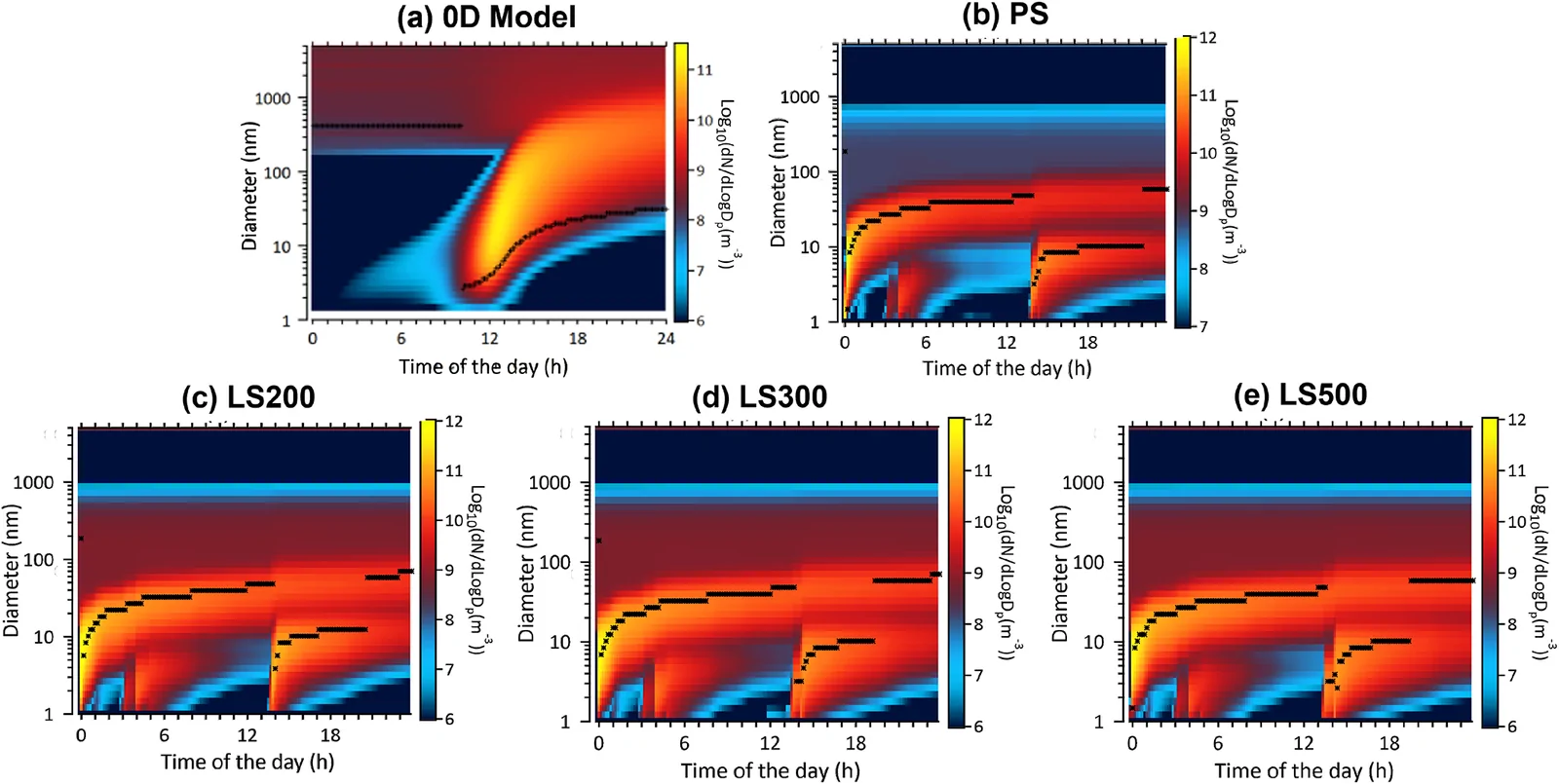
Time evolution of particle number size distributions (log_10_(*dN*/*d* log *D*_*p*_)) at the central grid point (30 m height) for the 0D reference model (panel a) and the four 3D emission cases (panels b–e). Color contours represent the logarithmic particle number concentration. The black line indicates the geometric mean diameter of the fitted nucleation mode (NPF mode) over time, obtained from mode fitting of the particle number size distributions.

In contrast, all four 3D simulations (panels b–e) depart from this idealized picture in the same qualitative way. Growth begins within the first few hours rather than at midday, the geometric mean diameter follows an intermittent rather than a smooth trajectory, and a second nucleation episode appears later in the day (around 13–14 h) as a fresh sub-10 nm mode. The nucleation mode also grows to larger sizes (of order 50–70 nm) than in the 0D case (∼30 nm). Notably, these features are common to all four emission geometries, which produce broadly similar banana morphologies at the observation point. In the present configuration, nucleation is evaluated in every grid cell that satisfies the local formation criteria (Supporting Section S2), so the central location continuously forms fresh particles from locally available precursors. As a result, the banana morphology is controlled primarily by the presence of resolved transport and local nucleation rather than by the specific source geometry. The recurring multiepisode structure arises because the evolving flow periodically advects larger particles away from the observation point, lowering the local condensation sink and allowing nucleation to resume (Supporting Figure S6.5). This behavior is absent in the 0D model, where the condensation sink remains uniformly high once particles have formed.

To test whether this similarity across geometries indeed stems from domain-wide nucleation, we repeated the simulations with the same conserved emission cases but with nucleation restricted to the emission source cells (Figure S6.8). Under this source-confined assumption the central observation point no longer forms its own particles and instead samples only the population transported in from the sources, and the geometries now diverge markedly. The point source and the smallest line source (PS, LS200) stabilize at moderate mean diameters (of order 50–70 nm), whereas the larger line sources (LS300, LS500) deliver substantially larger, more aged modes (reaching several hundred nm), as particles grow during their longer transport to the center. The recurring sub-10 nm bursts seen in Figure 6 are largely absent, consistent with the suppression of local nucleation away from the sources.

Beyond these spatial differences, the earlier onset of nucleation in the 3D cases is consistent with observations that particle formation is not confined to the period of peak midday photochemistry. In forested and biomass-burning environments, nocturnal formation and growth are often attributed to organic vapors. Mishra et al.,^6^ for instance, reported frequent growth events in New Delhi that began in the evening and persisted overnight, attributed to condensation of primary organic vapors despite low sulfuric acid. However, nighttime and early morning nucleation has also been reported in inorganic, low-organic systems. Nighttime sulfuric acid produced by nonphotochemical oxidation drives sub-3 nm cluster formation on clean, low-condensation-sink nights in urban Beijing,^26^ and ion-induced sulfuric-acid–ammonia nucleation sustains NPF in pristine coastal Antarctica.^27^ Across these environments the common enabling factor is a low condensation sink rather than enhanced precursor production; early morning NPF in the tropical free troposphere, for example, occurs under very low CS despite weak photochemistry.^28^ This is the regime our simulations reproduce: because precursor concentrations and the condensation sink vary in space, nucleation is governed by local rather than domain-averaged conditions. Figure S6.5 shows that the 3D cases nucleate early and in intermittent bursts even though their domain-median H_2_SO_4_ (∼10^6^–10^8^ m^–3^) is far below the 0D value (∼10^13^–10^14^ m^–3^). Early onset therefore arises from transient, localized low-CS conditions that allow nucleation thresholds to be reached at low domain-mean vapor, without invoking organic precursors.

In the real atmosphere, the earlier onset would likely be reinforced by processes that our configuration only partly represents. The simulated flow is nudged at the lateral boundaries to hourly observed winds, so the horizontal transport and its diurnal evolution reflect measured meteorological forcing rather than an idealized flow, and the spatial heterogeneity that drives early nucleation therefore emerges under observation-based conditions. The thermodynamic component of the diurnal cycle, however, is not represented: because temperature is prescribed as spatially uniform and buoyancy-driven stratification is neglected, the model does not reproduce the nocturnal stabilization of the boundary layer. In the atmosphere, after sunset surface radiative cooling stabilizes the boundary layer and suppresses vertical mixing, so that emitted vapors remain in a shallow layer with reduced dilution and are carried predominantly by horizontal winds, sometimes through nocturnal low-level jets.^29^ Together with overnight removal of pre-existing particles, such conditions tend to lower the condensation sink and favor nucleation at low vapor concentrations, consistent with the low-CS nocturnal events noted above.

These results show that banana-plot morphology at a fixed location reflects not only gas-phase chemistry and the timing of nucleation, but also where new particles form and how transport redistributes them. When nucleation occurs throughout the domain, local formation dominates the observed signal and largely overrides source geometry; when it is confined to emission regions, the observed size distribution is instead set by source extent and transport distance. In both cases the 3D morphologies differ substantially from the smooth 0D banana over separations of less than 1 km, a scale conventionally assumed to be environmentally homogeneous. Consequently, inferring NPF mechanisms or classifying event types from banana-plot morphology alone may be misleading, since local transport, mixing, and the spatial distribution of nucleation can substantially reshape the observed size distribution and apparent growth rates.

Figure 7 shows the time evolution of size-resolved aerosol number distributions (*dN*/*d* log *D*_*p*_) at the central grid point (30 m height) for the four 3D emission geometries and the 0D reference model, in successive 2 h intervals. This figure complements the banana plot in Figure 6 by resolving, in discrete time slices, how the mode structure of the size distribution evolves in each scenario.

**Figure 7:**
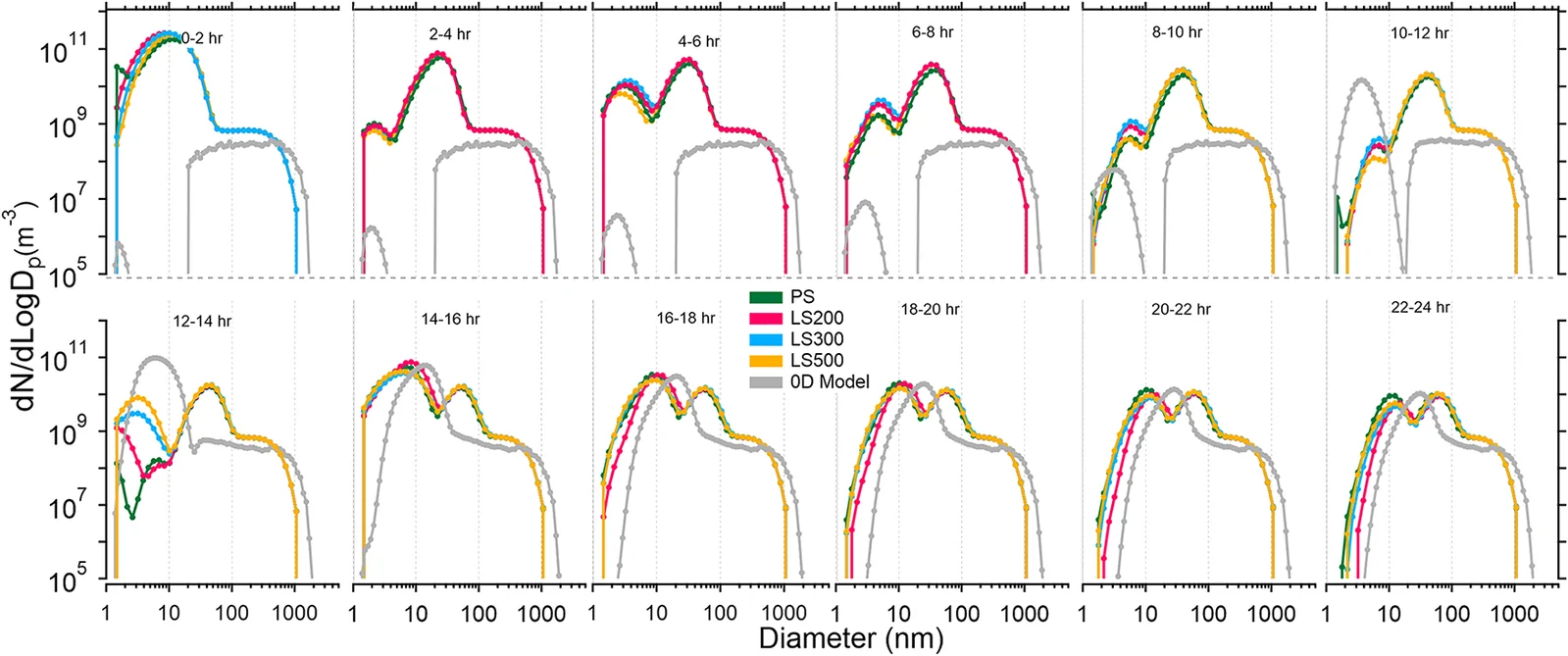
Time evolution of size-resolved aerosol number distributions (*dN*/*d* log *D*_*p*_) at the central grid point (30 m height) for the four 3D emission cases and the 0D reference model in varying colors. Each panel corresponds to a 2 h time interval over the 24 h simulation. Number concentrations are shown on a logarithmic scale to highlight both nucleation and accumulation modes.

Differences between the 3D and 0D results emerge within the first few hours. By 0–4 h, all four 3D cases already display a well-developed mode below ∼10 nm, whereas the 0D model shows little more than the prescribed background and does not begin to nucleate until around midday (10–14 h). This early divergence is consistent with the earlier onset seen in the banana plot and reflects the role of resolved transport and local nucleation in supplying and processing vapor at the observation point.

The most prominent feature of the 3D distributions is their persistent bimodality. From ∼4 h onward, the 3D cases develop two coexisting modes. These are a fresh mode near 5–10 nm and a grown mode near 40–60 nm, separated by a clear minimum around 15–25 nm. This structure recurs throughout the simulation and across all emission geometries, whereas the 0D model remains essentially unimodal at all times, evolving as a single broad mode. The contrast is mechanistically informative. A second, separate mode at a fixed point requires two particle populations with different histories to coexist there: a freshly formed or recently arrived mode together with an older, transported and aged mode. In a spatially homogeneous box model this cannot occur, because all particles share the same growth history. The bimodality is therefore a direct fingerprint of the 3D environment, in which transport continually brings together air parcels of different age, and it is the size-distribution counterpart of the multiepisode banana structure in Figure 6.

The interplay between the two frameworks is clearest near midday. Around 12–14 h the 0D model reaches its peak nucleation, appearing as a strong fresh mode near 3–5 nm, while at the same time the 3D cases show a pronounced minimum at these small sizes, their freshly formed particles having been diluted or advected away so that the grown mode near 50 nm dominates. The two frameworks are thus nearly out of phase at the smallest sizes at this time. Later in the simulation (18–24 h) the 0D and 3D distributions partially converge at larger sizes as the accumulation mode builds up, but the 3D cases retain a fresh sub-10 nm mode that the 0D model does not reproduce.

Overall, the time-resolved distributions confirm that the 3D framework captures mode-structure and timing variability the 0D model cannot. Because multimodal sub-100 nm distributions with a distinct minimum are also frequently observed in ambient measurements, such structure need not indicate multiple chemical mechanisms but can arise from transport and mixing acting on a single nucleation pathway.

Figure 8 compares the size-resolved growth rates of the four 3D emission scenarios and the 0D reference model against observed nanoparticle growth rates compiled from field studies across urban, rural, remote, coastal, and high-altitude sites (SI Table S6.5). For every framework the plotted GR is obtained by tracking the growing mode of the simulated size distribution at the central grid point over time (SI Figure S6.9). The 0D model systematically underpredicts growth across the size range, whereas the 3D scenarios reproduce both the magnitude and the size dependence of the observations.

**Figure 8:**
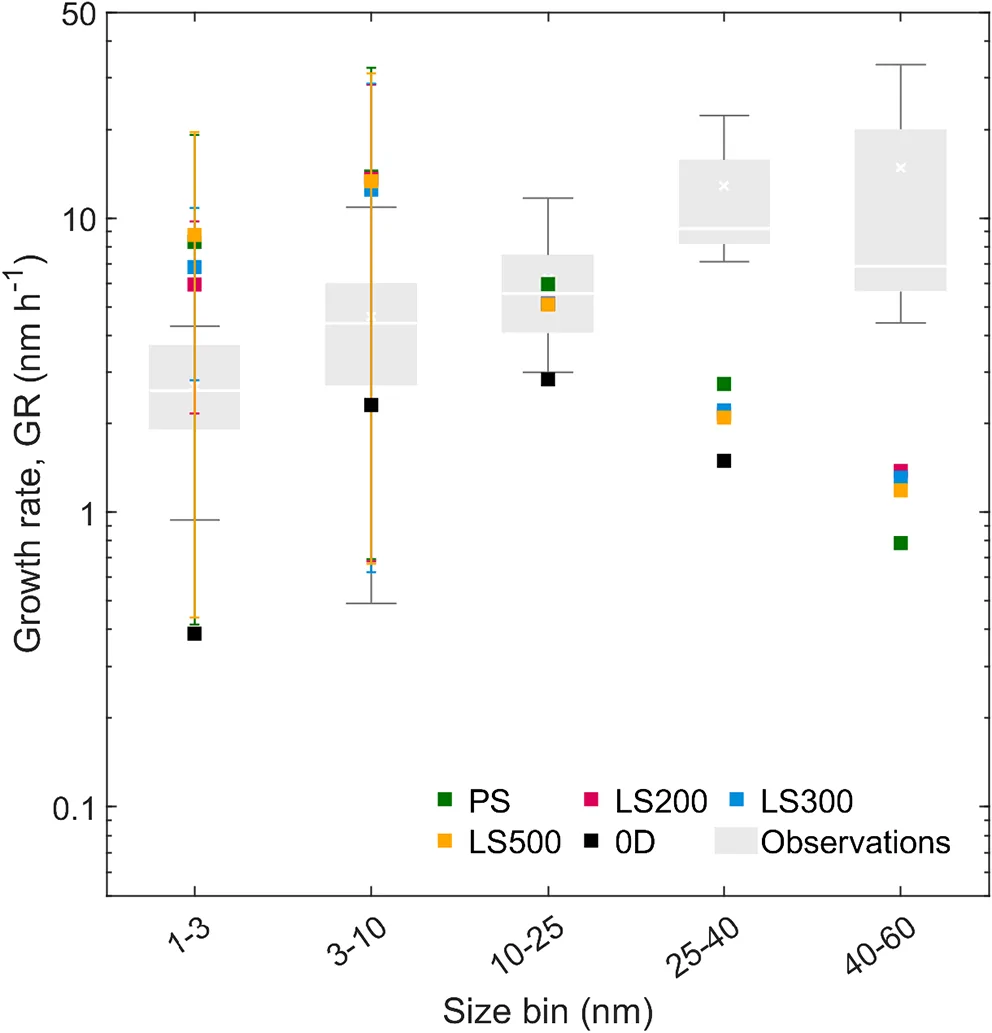
Size-resolved growth rates (GR, nm h^–1^) for the four 3D emission scenarios (PS, LS200, LS300, and LS500) and the 0D reference model, compared against observed nanoparticle growth rates across five diameter bins: 1–3, 3–10, 10–25, 25–40, and 40–60 nm. Modeled points represent event-based median GRs, derived by tracking the growing mode of each simulated size distribution over the 24 h period (SI Figure S6.9). Gray boxes summarize the field measurements mapped to each size bin, including medians, interquartile ranges, and whiskers, with means shown as crosses. The contributing studies are provided in SI Table S6.5.

The gap is largest in the 1–3 nm range, where newly formed particles are most vulnerable to loss and event survival is effectively decided. Here the 0D model grows the nucleation mode at only ∼0.4 nm h^–1^ (SI Table S6.1), roughly six times below the observed median (∼2.6 nm h^–1^), whereas the 3D scenarios grow it at ∼6–9 nm h^–1^, within the observed range once their event-to-event spread is accounted for (SI Table S6.2). This matters because the survival of freshly formed clusters is governed by the competition between growth and scavenging, expressed through the survival parameter *P* = (CS/ 10^–4^ s^–1^)/(GR/nm h^–1^), in which survival falls steeply as CS rises or GR falls.^30,31^ The 0D framework combines a high, spatially uniform condensation sink with these low growth rates, driving *P* into a regime of vanishing survival, whereas the 3D framework resolves localized microenvironments where vapor accumulates and the sink is locally reduced. At larger sizes the four emission geometries converge closely (e.g., ∼5.1–6.0 nm h^–1^ at 10–25 nm and ∼2.1–2.7 nm h^–1^ at 25–40 nm; SI Table S6.1), mirroring the overlapping banana plots and size distributions of Figures 6 and 7 and confirming that growth at these sizes is set by transport and microphysics rather than by source geometry; throughout, the 3D rates track the observed medians far more closely than the 0D model.

This bias originates in the homogeneity assumption itself: box representations distribute condensable vapor onto a single, domain-mean size distribution and implicitly assume a spatially extensive air mass in which nucleation and growth can be followed at one point.^31^ Real growth is instead enhanced by localized sources and “typically not observed at extended spatial scales”,^31^ so domain-mean fields miss the fast growth measured in the field. Some departures remain: in the 40–60 nm bin the modeled rates fall below the observations, consistent with vapor depletion and the absence of low-volatility organic condensation and hygroscopic growth here (see Implications and Outlook section). The comparison is therefore best read as evidence that resolving microscale transport captures the correct magnitude and size dependence of NPF growth, rather than as a fully quantitative match.

Because the two frameworks share identical chemistry, microphysics, and inputs and differ only in whether the environment is resolved, their ratio isolates the effect of spatial representation. At the survival-limiting sub-3 nm sizes, resolving the 3D environment raises the predicted growth rate from 0.39 nm h^–1^ to 6.0–8.8 nm h^–1^ (SI Table S6.1), an increase of roughly 15–23× (mean ∼ 20×). This divergence is largest at the smallest sizes and diminishes with diameter, the root-mean-square 3D–0D difference falling from ∼7 nm h^–1^ at 1–3 nm to ∼2.5 nm h^–1^ at 10–25 nm and ∼0.8 nm h^–1^ at 25–40 nm (SI Table S6.4). Neglecting meter-scale structure therefore biases predicted growth most severely at the sub-3 nm sizes that control particle survival, precisely where accuracy matters most for the particle number budget.

### Implications and Outlook

Our results demonstrate that meter-scale environmental structure strongly modulates when and where new particle formation occurs and how newly formed particles grow under realistic urban conditions. Even weak background winds sustain substantial heterogeneity in vapor convergence, dilution, and particle sinks, producing large variations in nucleation intensity, growth rates, and size-distribution morphology. This provides a physical contribution to the long-standing early growth discrepancy often attributed solely to missing chemical pathways. Because the 3D and 0D configurations differ only in whether the environment is resolved, their comparison isolates this effect: resolving spatial structure raises predicted growth at the survival-limiting sub-3 nm sizes by up to ∼20× and brings modeled rates within the range of field observations, whereas the homogeneous box model underpredicts across the size spectrum. Assuming homogeneity therefore biases growth-rate estimates and can misattribute size-distribution features such as banana-plot morphology and persistent bimodality to multiple chemical mechanisms, when they may instead arise from transport and mixing acting on a single nucleation pathway.

A key consequence is that large-scale models, which cannot resolve these structures, may misrepresent NPF when using grid-mean precursor concentrations, since the nonlinear dependence of nucleation and growth on vapor lets localized precursor-rich regions contribute disproportionately to total particle formation. This argues for representing subgrid precursor variability in regional and global models, analogous to subgrid treatments in cloud microphysics. The same heterogeneity may also bear on the persistent underprediction of particle number and cloud condensation nuclei (CCN) by large-scale models, usually attributed to incomplete nucleation and growth chemistry:^2,3^ microenvironments that raise sub-3 nm survival would enhance the number of particles reaching CCN sizes beyond what grid-mean conditions predict.

These conclusions rest on deliberately simplified chemistry and growth, adopted to isolate the role of transport. Sulfuric acid is prescribed and base vapors emitted uniformly, without interactive chemistry–transport feedbacks. The central result, that heterogeneity strongly influences nucleation and growth, does not depend on which precursor dominates the variability, but on the nonlinear dependence of NPF on local concentrations. In the real atmosphere sulfuric acid, being oxidation-produced, is often more spatially uniform than the locally emitted bases, so heterogeneity may reside more in the base field; because nucleation depends on both precursors and responds nonlinearly to their local concentrations, this would still generate the localized NPF variability found here, and may enhance it. The quantitative results are nonetheless specific to acid–base systems, and growth is underestimated at larger sizes because low-volatility organics and hygroscopic growth (κ = 0) are omitted (Figure 8), while the sub-3 nm, transport-controlled behavior that drives our conclusions is unaffected. The neglect of hygroscopic growth in particular decouples particle growth from relative humidity and omits water uptake by the acid–base particles, which are hygroscopic under atmospheric conditions, most plausibly contributing to the underestimation of growth beyond the nucleation mode. Beyond informing parametrization, 3D-ICAM thus provides a controlled platform that disentangles chemistry from environmental variability under identical inputs, which is not possible with box models and difficult from observations alone. Whether these findings extend to organic-driven NPF depends on the controlling regime: where organic NPF is precursor-limited it should behave as shown here, whereas where it is oxidant-limited the relevant heterogeneity may reside in the oxidant field rather than the emitted precursor, so that the response to microscale flow could differ in magnitude and structure. Future developments incorporating interactive chemistry, oxidant variability, organic vapors, hygroscopic growth, and boundary-layer stratification will extend it from process-level insight toward quantitative prediction of atmospheric NPF.

## Supplementary Material



## Data Availability

All simulation data and analysis scripts underlying the figures in this study are available via a public Zenodo repository (DOI: 10.5281/zenodo.17036552).

## Notes

Full mathematical formulations of the Air3D, AMChem, and Aero3D modules; input parameters; three-dimensional spatio-temporal evolution of the particle number concentration in the size range 10–25 nm; three-dimensional spatio-temporal evolution of sulfuric acid concentration (H_2_SO_4_, m^–3^); dependence of turbulent kinetic energy (TKE, m^2^ s^–2^) on grid spacing (Δ*x*); time evolution of particle number size distributions (log_10_(*dN*/*d* log *D*_p_)) at the central grid point (30 m height) for the 0D reference model (panel a) and the four 3D emission cases (panels b–e); event-based median growth rate (GR) in nm h^–1^; number of event GR values used after full-crossing preference; and size-resolved observational nanoparticle growth rates (GR) mapped to the model size bin (PDF)
