# Supplementary material for: How Microscale Flow and Vapor Transport Shape New Particle Formation and Growth
Source: Environ Sci Technol. 2026 Aug 17;60(40):28994–9005. doi: 10.1021/acs.est.6c10206 (PMC13674053; doi:10.1021/acs.est.6c10206)
Supplement: Supplementary file 1 [file es6c10206_si_001.pdf]

Supporting Information: How Microscale Flow and Vapor Transport Shape New Particle Formation and Growth

Kunal Ghosh,*,† Gargi Sengupta,‡ and Suneeti Mishra¶

†School of Earth, Environment and Sustainability, University of Leeds, Leeds, LS2 9JT, UK

‡Environmental Research Group, School of Public Health, Imperial College London, White City Campus, Wood Lane, London W12 0BZ, UK

¶PSI Center for Energy and Environmental Sciences, Villigen 5232, Switzerland

E-mail: k.ghosh@leeds.ac.uk

S1 Full Governing Equations for Air3D

Air3D is a three-dimensional extension of the two-dimensional CFD–aerosol dynamics model developed in Ghosh et al.[S1]. It solves the conservation equations for mass, momentum, energy, and species, using a finite-volume approach with a buoyancy-modified standard $k-\varepsilon$ turbulence closure scheme. The governing equations are:

- **Mass conservation (continuity):**

$$\underbrace{\frac{\partial \rho}{\partial t}}_{\text{local density change}} + \underbrace{\frac{\partial}{\partial x_i}(\rho u_i)}_{\text{advective transport}} = 0 \tag{S1.1}$$

where ρ is the air density, t is time, $x_i \in \{x, y, z\}$ denotes spatial direction, and u_i is the velocity component in the x_i direction. The first term represents the local change

in density with time, and the second term accounts for the divergence of mass due to advection.

- **Momentum conservation (including buoyancy):**

$$\begin{aligned}
&\underbrace{\frac{\partial(\rho u_i)}{\partial t}}_{\text{local change in momentum}} + \underbrace{\frac{\partial}{\partial x_j}(\rho u_i u_j)}_{\text{convective transport}} \\
&= \underbrace{-\frac{\partial P}{\partial x_i}}_{\text{pressure gradient}} + \underbrace{\frac{\partial}{\partial x_j}\left[\mu_{\text{eff}}\left(\frac{\partial u_i}{\partial x_j}+\frac{\partial u_j}{\partial x_i}\right)\right]}_{\text{viscous stress}} + \underbrace{\rho g_i}_{\text{buoyancy}}
\end{aligned} \tag{S1.2}$$

where P is pressure, $\mu_{\text{eff}} = \mu + \mu_t$ is the effective dynamic viscosity (molecular μ combined with turbulent μ_t), and g_i is the component of gravitational acceleration. Each term represents forces per unit volume acting on a fluid parcel.

- **Energy conservation:**

$$\begin{aligned}
&\underbrace{\frac{\partial(\rho T)}{\partial t}}_{\text{local temperature change}} + \underbrace{\frac{\partial}{\partial x_i}(\rho u_i T)}_{\text{advective heat transport}} \\
&= \underbrace{\frac{\partial}{\partial x_i}\left[\left(\frac{\mu}{\text{Pr}}+\frac{\mu_t}{\text{Pr}_t}\right)\frac{\partial T}{\partial x_i}\right]}_{\text{thermal diffusion}} + \underbrace{\frac{\partial Q_{\text{th}}}{\partial t}}_{\text{thermal source}}
\end{aligned} \tag{S1.3}$$

where T is temperature, Pr and Pr_t are molecular and turbulent Prandtl numbers, respectively. In this study, we assume that emissions are at ambient temperature and do not contribute additional heat; hence, the change in thermal energy with time $\frac{\partial Q_{\text{th}}}{\partial t} = 0$.

- **Species transport:**

$$\underbrace{\frac{\partial(\rho C)}{\partial t}}_{\text{local concentration change}} + \underbrace{\frac{\partial}{\partial x_i}(\rho u_i C)}_{\text{advective transport}} = \underbrace{\frac{\partial}{\partial x_i}\left[\left(\frac{\mu}{\mathrm{Sc}} + \frac{\mu_t}{\mathrm{Sc}_t}\right)\frac{\partial C}{\partial x_i}\right]}_{\text{diffusion (molecular + turbulent)}} + \underbrace{S_C}_{\text{source}} - \underbrace{4J_{A4B4}}_{\text{nucleation sink}} \tag{S1.4}$$

where C is the vapor concentration, Sc and Sc_t are the molecular and turbulent Schmidt numbers, S_C is the source term representing emissions or chemical production of the species, and J_{A4B4} is the nucleation rate (Section S2, Eq. S2.2). Since each A4B4 cluster comprises four acid and four base molecules, the sink is applied with a stoichiometric factor of four to sulfuric acid and, for the base, to dimethylamine (DMA), which dominates cluster formation as it gives the highest nucleation rate of the three bases at a given H_2SO_4 concentration (Fig. S4.1b); consumption via the weaker MEA and NH_3 channels is not tracked separately. Both species are subject to the nucleation sink while being supplied by their prescribed sources, so their concentrations reflect the local balance between supply and nucleation loss.

Air3D provides dynamically evolving 3D fields of temperature, humidity, velocity, and pressure to AMChem at each time step. These fields modulate nucleation, condensation, and gas–particle chemistry. Updated vapor fields from AMChem feed back into the species transport equation in Air3D. Coupling is performed using a fully implicit scheme to maintain stability under sharp gradients.

S2 Full Governing Equations for AMChem

The AMChem module simulates aerosol dynamics through nucleation, coagulation, condensation, and deposition using a sectional representation of particle sizes. The general dynamic equation for the number concentration $N(V, x_i, t)$ of particles with volume V, at spatial lo-

cation $x_i \in \{x, y, z\}$ and time t, is given by:

$$\begin{aligned}
\frac{\partial N(V, x_i, t)}{\partial t} = & \underbrace{J_{A4B4}(x_i, t)\, \delta(V - V_{\text{crit}})}_{\text{nucleation}} \\
& + \underbrace{\frac{1}{2} \int_0^V K_g(V', V - V')\, N(V', x_i, t)\, N(V - V', x_i, t)\, dV'}_{\text{coagulation production}} \\
& - \underbrace{N(V, x_i, t) \int_0^\infty K_g(V, V')\, N(V', x_i, t)\, dV'}_{\text{coagulation loss}} \\
& - \underbrace{\frac{\partial}{\partial V} \left[C(V, x_i, t)\, N(V, x_i, t) \right]}_{\text{condensational growth}} \\
& - \underbrace{k(V, x_i, t)\, N(V, x_i, t)}_{\text{deposition loss}}
\end{aligned} \tag{S2.1}$$

Each term in Equation S2.1 represents a physical process:

- **Nucleation term**:

  This term describes the formation of stable clusters from gas-phase precursors, implemented using the cluster-kinetic formulation from Brean et al.[S2]. For each base species b, the four-acid–four-base nucleation rate is given by:

$$\begin{aligned}
J_{A4B4}^{(b)} = & \frac{\beta_{A_1B_1}^3}{2\left(CS + \beta_{A_1B_1}[H_2SO_4]\right)^2} \, [H_2SO_4]^4 \, \eta^4 \\
& \times \left[1 + 2 \frac{\gamma + CS}{\beta_{A_1B_1}[B_b] + CS} \right] \\
& \times \left[1 + \frac{1}{4} \left(1 + 2 \frac{\gamma + CS}{\beta_{A_1B_1}[B_b] + CS} \right) \right]
\end{aligned} \tag{S2.2}$$

  where $[H_2SO_4]$ and $[B_b]$ represent the gas-phase concentrations of sulfuric acid and the participating base species b, respectively, such as DMA, MEA, or NH_3. The parameter $\beta_{A_1B_1}$ denotes the collision rate of acid–base dimers, while γ is the evaporation

rate of intermediate molecular clusters. The term CS represents the condensation sink, accounting for vapor losses to pre-existing particles (see supporting information in Brean et al.[S2]). Finally, η is the cluster formation efficiency, reflecting the kinetic probability that colliding acid–base molecules proceed to form a stable cluster. The total nucleation rate entering Eq. S2.1 is obtained by summing the base-specific rates, $J_{\mathrm{A4B4}} = \sum_b J^{(b)}_{\mathrm{A4B4}}$; DMA dominates this sum (Fig. S4.1b). Because the nucleation rate in Eq. (S2.2) depends only on local quantities, it is evaluated independently in each grid cell, and new-particle formation proceeds wherever these conditions yield a non-zero rate. This is consistent with the standard treatment of nucleation as a local source term in spatially resolved aerosol models, including high-resolution CFD- and LES-coupled aerosol frameworks[S3,S4], in which clusters form wherever precursors are transported and the formation thresholds are met, rather than only at emission locations. The framework also supports a source-confined configuration, in which Eq. (S2.2) is evaluated only within the emission source cells. This represents the limiting assumption that particle formation is co-located with emission, with particles elsewhere arising solely through transport and growth. We use this configuration as a sensitivity case (Fig. S6.8); unless stated otherwise, all results correspond to the spatially unrestricted configuration.

- **Coagulation terms**:

These terms represent particle–particle collisions across five different coagulation pathways. The first integral corresponds to the gain of particles of volume V from collisions of smaller particles, while the second represents the loss of particles of volume V due to collisions with other particles of volume V'. In this study, we include the Brownian coagulation kernel ($b^{B}_{V,V'}$), which accounts for Brownian motion–driven collisions with transition-regime corrections; the convective Brownian diffusion enhancement kernel ($b^{DE}_{V,V'}$), which incorporates enhancement of Brownian coagulation by convective motion; the gravitational collection kernel ($b^{GC}_{V,V'}$), representing collisions due to differences

in terminal settling velocities including collision efficiency effects; the turbulent inertial motion kernel ($b^{TI}_{V,V'}$), describing coagulation from inertial impaction in turbulent flows; and the turbulent shear kernel ($b^{TS}_{V,V'}$), which accounts for shear-induced collisions in turbulent flow. All kernels are implemented following the formulations in Vohra et al.[S5], which include slip-flow corrections and Van der Waals interactions where applicable. The total coagulation kernel is then given by

$$K_g(V, V') = b^B_{V,V'} + b^{DE}_{V,V'} + b^{GC}_{V,V'} + b^{TI}_{V,V'} + b^{TS}_{V,V'}. \tag{S2.3}$$

For the nucleation and early-growth size ranges emphasized in this study (1–40 nm), Brownian coagulation dominates direct particle–particle collisions, as the Brownian kernel is large for nanometre-sized particles while the gravity- and turbulence-related kernels are comparatively small at these sizes. These additional kernels are retained because the sectional model resolves the full aerosol size distribution from 1 to 5000 nm, including larger pre-existing particles whose number and size distribution can be affected by gravitational collection, turbulent inertial motion, and turbulent shear. These larger particles contribute substantially to the condensation sink; the additional kernels therefore influence nucleation and early growth primarily indirectly, through their effect on the background aerosol population and hence on the condensation sink that governs vapour availability and particle survival.

Condensational growth term: Condensational growth is represented as a flux in particle-volume space. In the general dynamic equation, $C(V, \mathbf{x}, t)$ denotes the single-particle volume growth rate, dV/dt, rather than a number-concentration tendency. For a particle of diameter D_p and volume $V = \pi D_p^3/6$, growth by sulfuric-acid condensation

is

$$C(V,\mathbf{x},t) = \frac{dV}{dt} = \frac{M_{\mathrm{H_2SO_4}}}{\rho_p N_A}\, 2\pi D_p D_{\mathrm{H_2SO_4}}\, \beta_m(D_p)\, \big([\mathrm{H_2SO_4}](\mathbf{x},t) - [\mathrm{H_2SO_4}]_{\mathrm{eq}}(D_p)\big), \tag{S2.4}$$

where $M_{\mathrm{H_2SO_4}}$ is the molar mass of sulfuric acid, ρ_p the particle density, N_A Avogadro's number, $D_{\mathrm{H_2SO_4}}$ the gas-phase diffusivity of sulfuric acid, and $\beta_m(D_p)$ the transition-regime correction. $[\mathrm{H_2SO_4}](\mathbf{x},t)$ is the local gas-phase sulfuric-acid concentration and $[\mathrm{H_2SO_4}]_{\mathrm{eq}}(D_p)$ the equilibrium vapour concentration over a particle of diameter D_p. Sulfuric acid is treated as the condensing vapour and charge enhancement is neglected. The transition-regime correction uses the Fuchs–Sutugin form

$$\beta_m(D_p) = \frac{1+Kn}{1+\left(\frac{4}{3\alpha}+0.377\right)Kn + \frac{4}{3\alpha}Kn^2}, \qquad Kn = \frac{2\lambda_{\mathrm{H_2SO_4}}}{D_p}, \tag{S2.5}$$

where $\lambda_{\mathrm{H_2SO_4}}$ is the mean free path of sulfuric acid vapour in air and α the mass accommodation coefficient. Equation (S2.4) has units of $\mathrm{m^3\,s^{-1}}$, as required for a volume growth rate; the corresponding diameter growth rate is

$$\frac{dD_p}{dt} = \frac{2}{\pi D_p^2}\frac{dV}{dt}. \tag{S2.6}$$

In the sectional implementation, condensational growth enters the general dynamic equation through the divergence term $-\partial[C(V,\mathbf{x},t)N(V,\mathbf{x},t)]/\partial V$, which transfers particle number between adjacent volume bins and has units of number concentration per unit time. Thus $C(V,\mathbf{x},t)$ is the single-particle growth rate, while $C(V,\mathbf{x},t)N(V,\mathbf{x},t)$ is the population flux in particle-volume space. The growth rates reported in the manuscript are diagnosed from the temporal evolution of the fitted growing mode (Section S5).

- **Deposition term**:

This term accounts for the removal of particles by dry deposition to the ground. Although written within the general dynamic equation (Eq. S2.1) as a size-resolved loss term for completeness, deposition is applied only at the lower (ground) boundary of the domain. Everywhere else the deposition term is set to zero, so that in the interior Eq. S2.1 reduces to nucleation, coagulation, and condensational growth alone, and deposition enters only as a surface flux at the ground. The deposition rate $k(V, x_i, t)$ is defined as in Vohra et al.[S5] and Ghosh et al.[S1], incorporating gravitational settling, surface interaction, and size-dependent deposition velocities.

All terms in AMChem are resolved at each time step using a semi-implicit numerical scheme to maintain stability and conserve mass and number as described in Ghosh et al.[S6]. The module receives updated environmental fields (temperature, RH, vapor concentrations) from Air3D and passes aerosol size distributions to Aero3D for 3D particle transport.

S3 Full Governing Equations for Aero3D

The Aero3D module tracks the evolution of the aerosol number concentration $N(V, x_i, t)$ as a function of particle volume V, spatial position $x_i \in \{x, y, z\}$, and time t. Transport occurs by advection and turbulent diffusion using flow and turbulence fields provided by Air3D.

The size-resolved transport equation, derived from the general dynamic equation in Ghosh et al.[S1], is:

$$\begin{aligned} \frac{\partial N(V, x_i, t)}{\partial t} \;+\; &\underbrace{\frac{\partial}{\partial x_i}\left[u_i(x_i, t)\, N(V, x_i, t)\right]}_{\text{advection}} \\ &= \underbrace{\frac{\partial}{\partial x_i}\left[D_{\text{eff}}(V, x_i, t)\,\frac{\partial N(V, x_i, t)}{\partial x_i}\right]}_{\text{turbulent + Brownian diffusion}} \end{aligned} \tag{S3.1}$$

Here, the term $\partial N/\partial t$ represents the local rate of change in aerosol number concentration. The velocity component $u_i(x_i, t)$ is the airflow in the x_i direction, obtained directly

from Air3D. The effective diffusivity $D_{\text{eff}}(V, x_i, t)$ combines Brownian diffusion, D_B, and turbulence-induced eddy diffusivity, D_t, such that

$$D_{\text{eff}} = D_B + D_t.$$

Brownian diffusion is computed using the Stokes–Einstein relation, while eddy diffusivity is derived from local turbulence intensity resolved by Air3D.

Aero3D operates on the size-resolved aerosol populations produced by AMChem at each time step and evolves them through the domain using the resolved flow and mixing fields. This allows explicit representation of spatial gradients and heterogeneity in aerosol evolution, features that cannot be captured by traditional 0D box models such as Brean et al.[S2], which assume a spatially homogeneous particle field.

S4 Input parameters

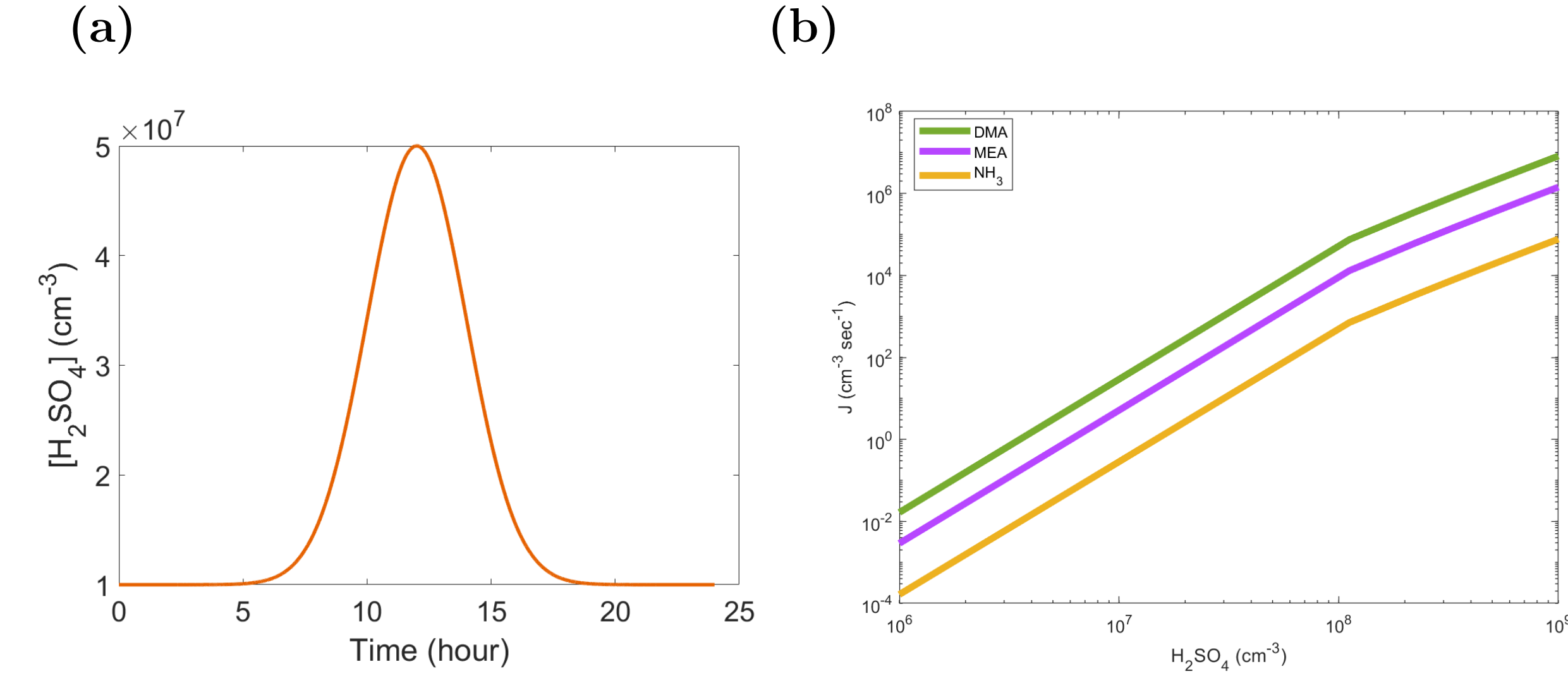

Figure S4.1: Chemical input parameters used in the simulations: (a) Diurnal variation of sulfuric acid concentration, peaking at noon with a maximum concentration of 5×10^7 cm^{-3}, representing photochemically driven production under clear-sky conditions: (b) Nucleation rates (J) as a function of sulfuric acid concentration for the three bases: dimethylamine (DMA), monoethanolamine (MEA), and ammonia (NH_3)[S2].

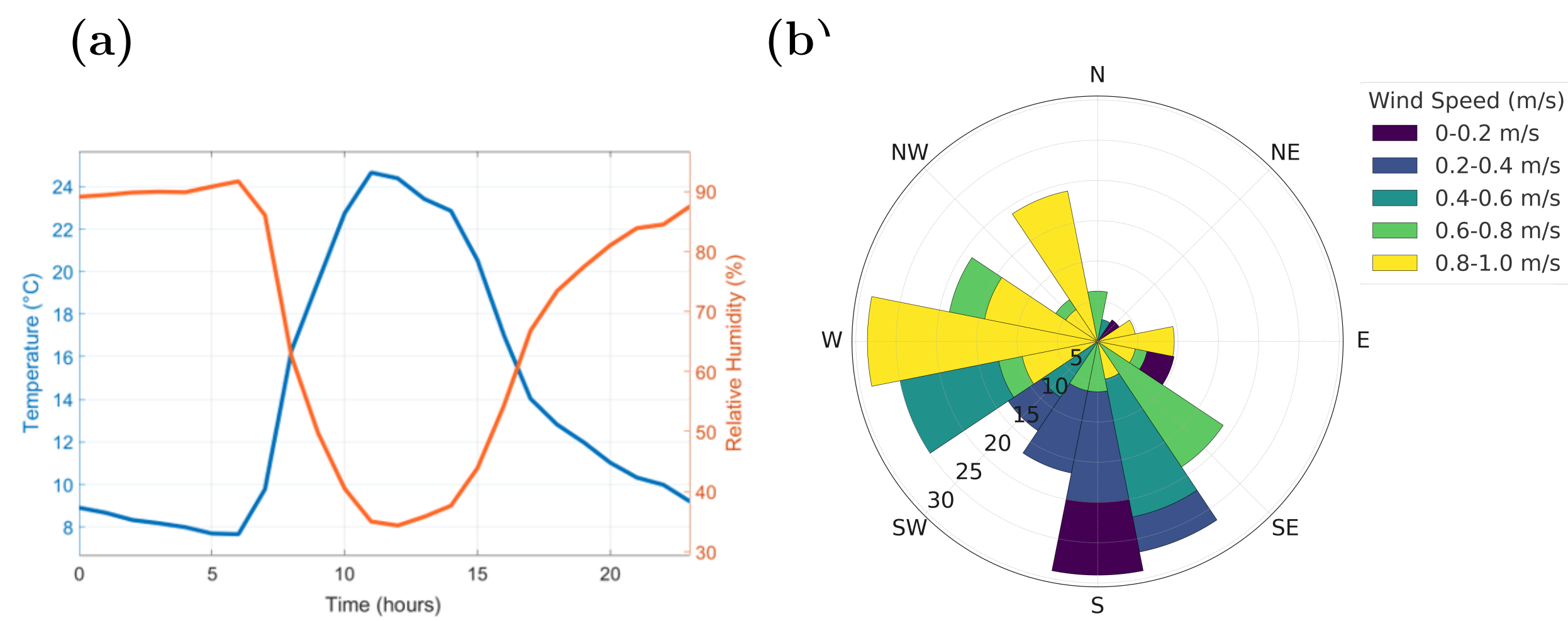

Figure S4.2: Meteorological input parameters used in the simulations, based on measurements reported in:[S7] (a) Diurnal cycle of temperature (blue, left axis) and relative humidity (orange, right axis), representing typical summertime urban conditions; (b) Wind rose diagram showing the distribution of wind direction and speed for the 24-hour period, with prevailing winds from the west and south and speeds mostly below 1 m s^{-1}.

S4.1 Meteorological and Emissions Forcing

Time-varying meteorological forcing is based on measurements reported by Mishra et al.[S7]. Hourly temperature, relative humidity, and horizontal wind speed and direction are used as time-dependent boundary conditions. The observed conditions correspond to weak-wind regimes, with horizontal wind speeds typically below $0.5\,\mathrm{m\,s^{-1}}$.

Wind velocity fields are prescribed at the lateral boundaries and nudged hourly toward the observed wind vectors to maintain realistic advection patterns (SI Fig. S4.2b). Temperature and relative humidity evolve in time according to the observed diurnal cycle (SI Fig. S4.2a).

Gas-phase precursors include H_2SO_4 and three bases: DMA, MEA, and NH_3. Base emission rates are held constant at 10^7 molecules $\mathrm{cm^{-3}\,h^{-1}}$, following Brean et al.[S2]; photochemical loss of amines (e.g., reaction with OH) is not explicitly represented, ensuring consistency with the 0-D reference framework. H_2SO_4 concentrations are prescribed as a time-varying profile with daytime maxima of order 10^7 molecules $\mathrm{cm^{-3}}$, based on Cai et al.[S8]. The diurnal evolution of H_2SO_4 and the resulting nucleation rates for each base combination are shown in SI Fig. S4.1.

To enable a physically consistent comparison between different emission geometries, the total precursor input into the domain is constrained to be identical across all cases. In the model, the precursor source term enters the species transport equation (Eq. S1.4) through S_C. A time-dependent emission strength $\Phi(t)$ is prescribed and applied only within the source region. The emission rate per grid cell is then scaled according to the total volume of the source region:

$$S_C^*(\mathbf{x},t) = \Phi(t) \times \frac{\Omega_{\mathrm{ref}}}{\Omega_{\mathrm{src}}} \quad \text{for } \mathbf{x} \in \Omega_{\mathrm{src}}, \tag{S4.1}$$

$$S_C^*(\mathbf{x},t) = 0 \quad \text{for } \mathbf{x} \notin \Omega_{\mathrm{src}}, \tag{S4.2}$$

where Ω_{src} is the total volume of the emission region and Ω_{ref} is the reference volume

corresponding to a single grid cell.

This formulation ensures that the total emission integrated over the source region remains constant,

$$\sum_{\mathbf{x}\in\Omega_{\mathrm{src}}} S_C^*(\mathbf{x},t)\,\Delta\Omega = \Phi(t)\,\Omega_{\mathrm{ref}}, \tag{S4.3}$$

which is independent of the spatial extent of the emission geometry.

As a result, line source configurations (LS200, LS300, LS500) have proportionally lower emission rates per grid cell compared to the point source, while maintaining the same total precursor input mass. This allows differences in particle number concentrations and growth rates to be attributed to emission geometry and transport processes, rather than variations in total emissions.

S4.2 Initial and Boundary Conditions of the Flow Field

Temperature and relative humidity are assumed to be spatially homogeneous but time-varying, following the prescribed diurnal cycles (SI Fig. S4.2a). Buoyancy forcing associated with horizontal temperature gradients is therefore not represented in the present configuration. Flow variability is instead dominated by mechanically driven processes, including horizontal advection and shear-induced turbulence, with turbulent mixing represented using a standard k–ε closure.

A parabolic vertical profile of horizontal wind speed is initialized above 10 m height to represent near-surface shear. In addition, a uniform vertical velocity of $w = 0.1$ m s^{-1} is applied only as an initial condition to establish the flow field. The velocity field thereafter evolves dynamically through the solution of the momentum equations, allowing spatial variations in velocity, shear, and turbulence to develop within the domain. A free-slip boundary condition is imposed at the top of the domain to allow vertical development of the flow without artificial damping.

S5 Model outputs

For each simulation, 3D-ICAM produces time-resolved three-dimensional fields at the native 10 m resolution: the size-resolved particle number distribution ($dN/d\log D_p$) in all 80 bins, together with the wind velocity, sulfuric acid concentration, nucleation rate J, and condensation sink. From these we derive size-resolved number concentrations (N_{1-3}, N_{3-10}, N_{10-25}), spatial fields at fixed times, central-grid-point time series for comparison with the 0D box model, and domain-averaged quantities with their spatial percentile ranges.

Growth rates are calculated from the temporal evolution of the size distribution. At each saved time step we fit the growing (NPF) mode and track its geometric mean diameter; the growth rate for a given size range is obtained from the rate at which the tracked mode traverses that range, in $\mathrm{nm\,h^{-1}}$. Growth rates are reported only where a mode crossing is detected. These population-based estimates integrate condensation, coagulation, continuous nucleation, and transport-driven variability in vapour availability, rather than idealised single-particle growth, and are consistent with the modal tracking used in observational studies[S7,S9]. In the 3D simulations the calculation is applied independently in each grid cell, yielding the local growth-rate fields of Fig. 5, while the same procedure applied to detected growth events provides the event-based growth rates used against the 0D model and observations (Fig. 8; SI Fig. S6.9, Table S6.1).

S6 Additional results

S6.1 Formation and distribution of newly formed particles (N_{3-10} and N_{10-25})

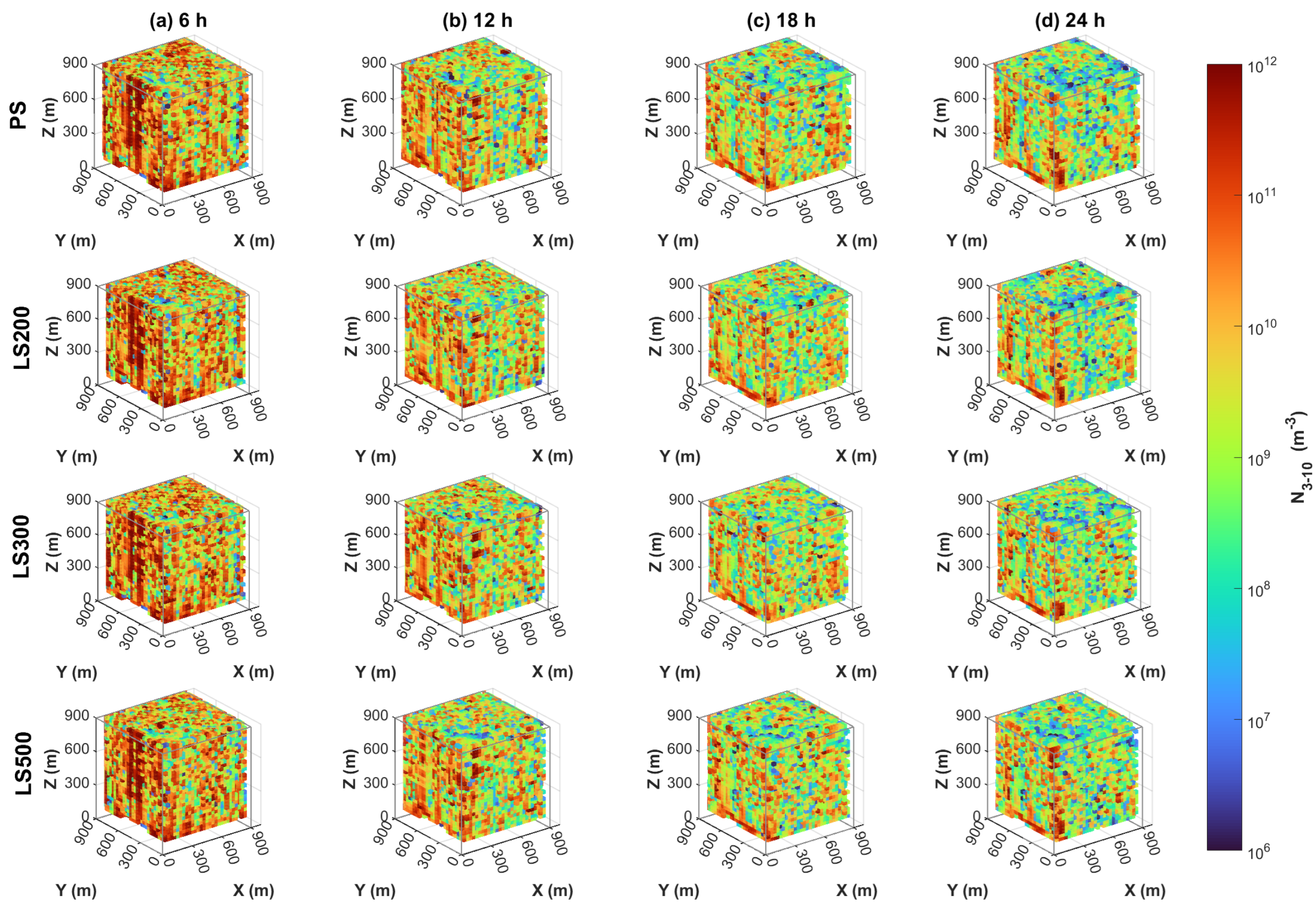

Figure S6.1: Three-dimensional spatio-temporal evolution of the particle number concentration in the size range 3−10 nm (N_{3-10}, m^{-3}) at (a) 6 h, (b) 12 h, (c) 18 h, and (d) 24 h. Colours represent $\log_{10}$-scaled concentrations. Rows correspond to different emission geometries: point source (PS), and line sources with characteristic lengths of 200 m (LS200), 300 m (LS300), and 500 m (LS500).

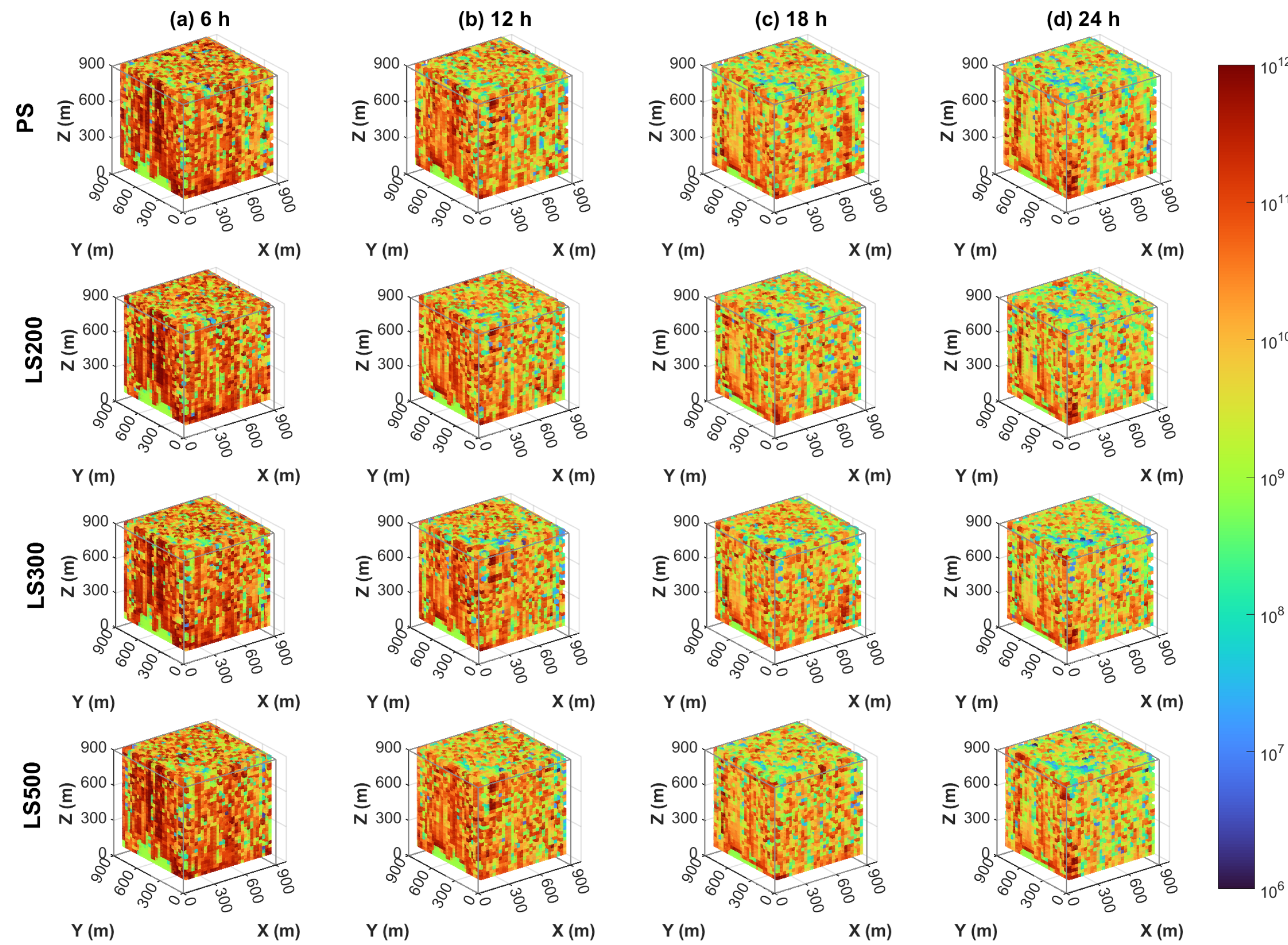

Figure S6.2: Three-dimensional spatio-temporal evolution of the particle number concentration in the size range 10−25 nm (N_{10-25}, m^{-3}) at (a) 6 h, (b) 12 h, (c) 18 h, and (d) 24 h. Colours represent $\log_{10}$-scaled concentrations. Rows correspond to different emission geometries: point source (PS), and line sources with characteristic lengths of 200 m (LS200), 300 m (LS300), and 500 m (LS500).

S6.2 Spatial structure of H_2SO_4 concentration and nucleation rate

To evaluate whether the sharp spatial gradients in particle number concentration and growth rate reported in the main manuscript arise from physical processes or numerical artefacts, we examine the corresponding three-dimensional distributions of sulfuric acid (H_2SO_4) concentration and nucleation rate (J).

Figure S6.3 shows the spatio-temporal evolution of H_2SO_4 concentration across the simulation domain, while Fig. S6.4 presents the corresponding nucleation rate fields. Both variables exhibit pronounced spatial heterogeneity, with localized regions of enhanced vapor

concentration and nucleation intensity forming under the influence of the evolving flow field.

Importantly, the locations of elevated particle number concentration and growth rate in the main manuscript (Figs. 4–5) are spatially consistent with regions of high H_2SO_4 concentration and nucleation rate shown here. This spatial co-location indicates that the gradients in particle properties are directly linked to physically resolved vapor convergence and dilution processes.

The coherence across precursor, nucleation, and particle fields demonstrates that the observed heterogeneity is dynamically generated and persists over time, rather than arising from transient numerical noise or discretization effects.

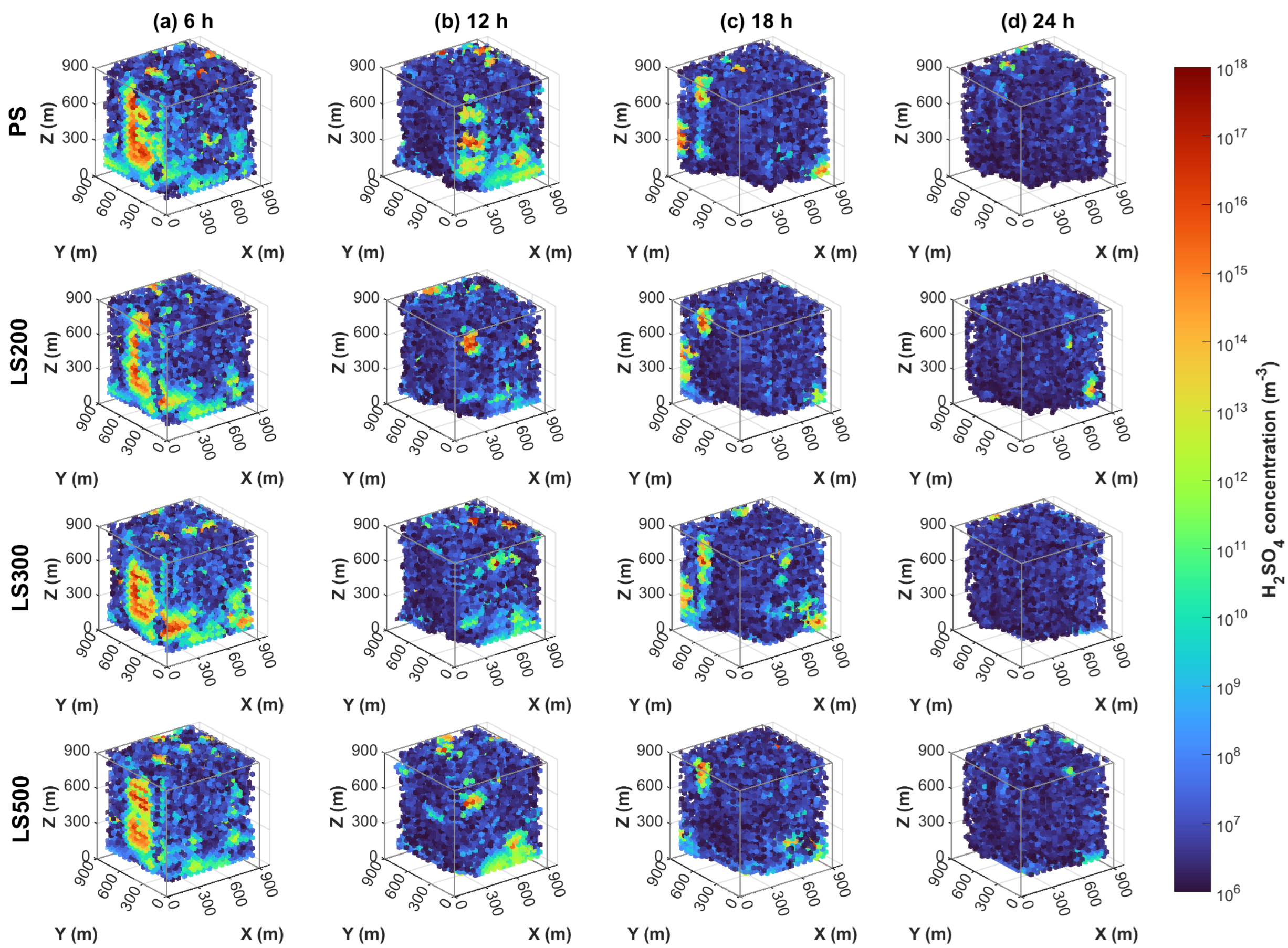

Figure S6.3: Three-dimensional spatio-temporal evolution of sulfuric acid concentration (H_2SO_4, m^{-3}) at (a) 6 h, (b) 12 h, (c) 18 h, and (d) 24 h. Colours represent $\log_{10}$-scaled concentrations. Rows correspond to different emission geometries: point source (PS), and line sources with characteristic lengths of 200 m (LS200), 300 m (LS300), and 500 m (LS500). The full computational domain (0–900 m) is shown in each panel.

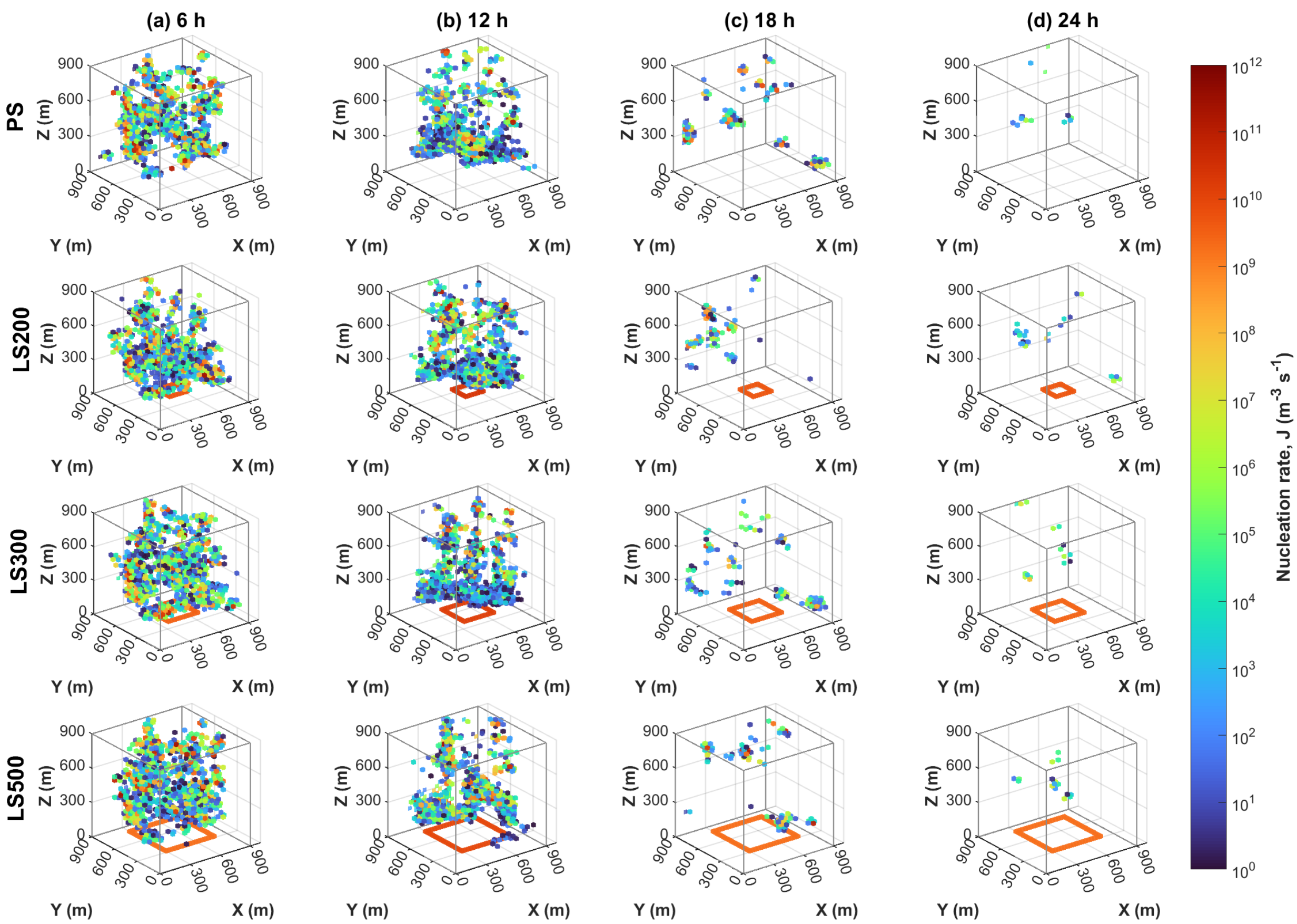

Figure S6.4: Three-dimensional distribution of the nucleation rate (J, $\mathrm{m^{-3}\,s^{-1}}$) at (a) 6 h, (b) 12 h, (c) 18 h, and (d) 24 h. Colours indicate $\log_{10}$-scaled values. Only grid cells with non-zero nucleation are shown. Rows correspond to different emission geometries: point source (PS), and line sources with characteristic lengths of 200 m (LS200), 300 m (LS300), and 500 m (LS500). The full computational domain (0–900 m) is indicated, with the emission region highlighted near the surface.

S6.3 Temporal evolution of domain-averaged quantities and comparison with the 0D model

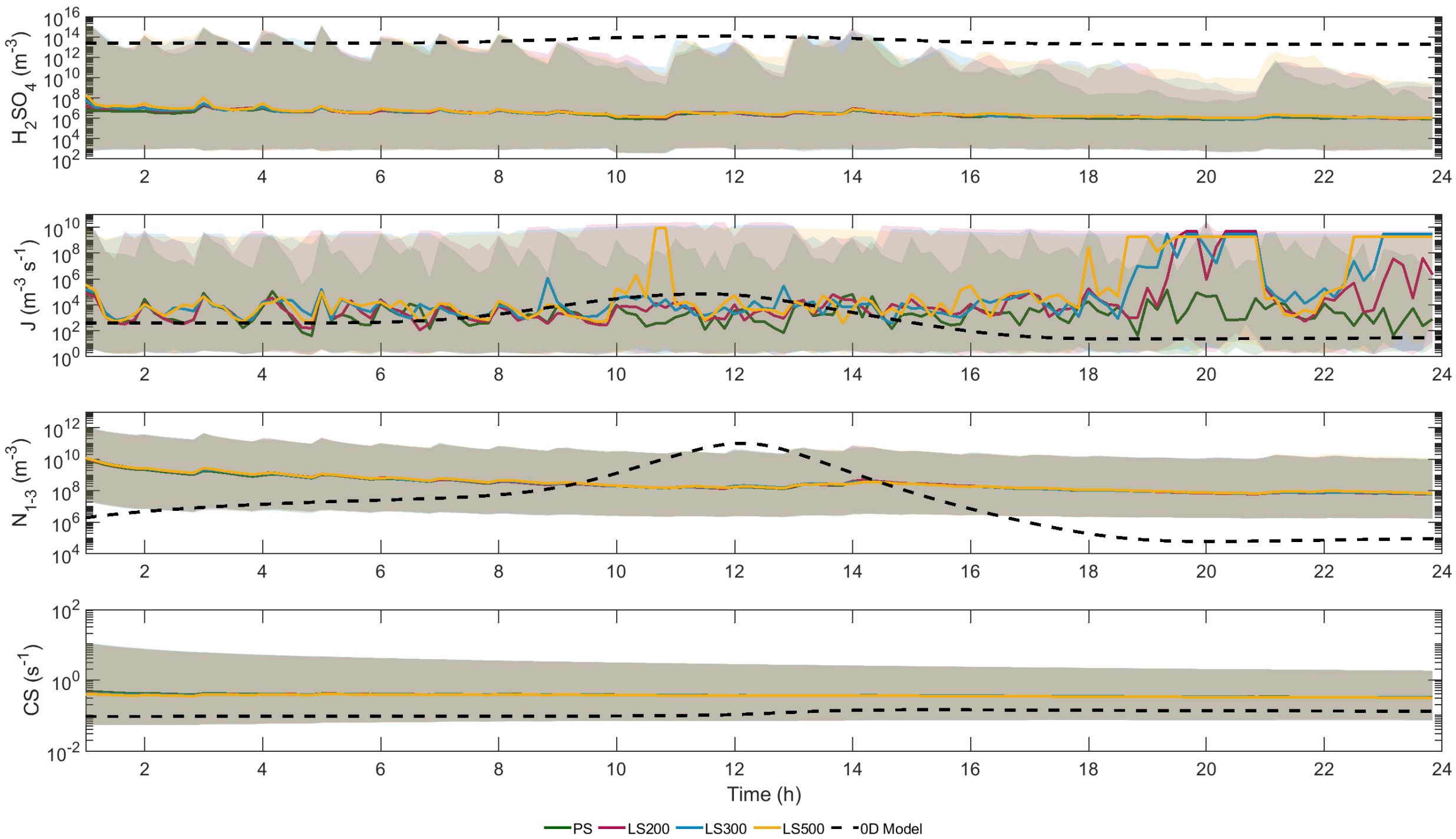

Figure S6.5: Temporal evolution of domain-averaged quantities for different emission geometries: point source (PS), and line sources with characteristic lengths of 200 m (LS200), 300 m (LS300), and 500 m (LS500), compared with the corresponding zero-dimensional (0D) model (black dashed line). Panels show (from top to bottom): sulfuric acid concentration (H_2SO_4, m^{-3}), nucleation rate (J, $m^{-3}\,s^{-1}$), number concentration of particles in the 1−3 nm size range (N_{1-3}, m^{-3}), and condensation sink (CS, s^{-1}). Shaded regions indicate the spatial variability (5th–95th percentile range) across the three-dimensional domain, while solid lines represent the domain median.

We examined the temporal evolution of sulfuric acid concentration, nucleation rate, nucleation-mode particle number concentration (N_{1-3}), and condensation sink in the 0D and 3D simulations (Fig. S6.5). The sulfuric acid concentrations (top panel) differ markedly between the 0D and 3D cases. In the 0D model, H_2SO_4 remains relatively high throughout the day, at $\sim 10^{13}$–$10^{14}\,m^{-3}$, with a maximum near midday following the prescribed diurnal profile. In contrast, the 3D simulations (PS and LS cases) exhibit much lower domain-median concentrations, generally in the range $\sim 10^{6}$–$10^{8}\,m^{-3}$, with intermittent fluctuations during the

day. The shaded percentile ranges indicate substantial spatial variability in the 3D fields, with localized regions reaching much higher sulfuric acid concentrations than the domain median.

The nucleation rate (second panel) shows a clear contrast between the two frameworks. In the 0D model, nucleation remains low during the early hours, increases gradually from around $\sim 8\,\mathrm{h}$, and reaches a peak near midday of order 10^4–$10^5\,\mathrm{m^{-3}\,s^{-1}}$, followed by a decline. In contrast, the 3D simulations exhibit strongly intermittent nucleation behavior, with multiple bursts occurring throughout the day, including during the early hours (1–4 h) and again during later periods. These early-time peaks indicate that nucleation can occur well before the 0D midday maximum when local conditions become favorable.

This difference is reflected in the evolution of N_{1-3} (third panel). In the 0D case, N_{1-3} increases from $\sim 10^6\,\mathrm{m^{-3}}$ during the early hours to a peak of order $10^{11}\,\mathrm{m^{-3}}$ near midday, before declining as particles grow out of the nucleation size range. In contrast, the 3D simulations exhibit elevated N_{1-3} already during the early hours, with median values of order $10^{10}\,\mathrm{m^{-3}}$ at $\sim 1\,\mathrm{h}$, followed by a gradual decrease to values of order $10^8\,\mathrm{m^{-3}}$ later in the day. This behavior reflects sustained contributions from both local nucleation bursts and transport of nucleation-mode particles.

The condensation sink (bottom panel) further highlights the differences between the two cases. In the 0D model, the condensation sink remains relatively uniform, increasing from approximately $0.095\,\mathrm{s^{-1}}$ in the early hours to about $0.14\,\mathrm{s^{-1}}$ later in the day. In contrast, the 3D simulations exhibit higher median condensation sink values, approximately 0.3–$0.5\,\mathrm{s^{-1}}$, together with substantial spatial variability, as indicated by the shaded percentile range. This variability implies the coexistence of regions with both enhanced and reduced scavenging efficiency within the domain.

These results show that the earlier and intermittent nucleation observed in the 3D simulations is not driven by higher domain-median sulfuric acid concentrations, which are much lower than in the 0D case. Instead, it arises from spatial heterogeneity in both vapor concen-

trations and condensation sink, which creates localized regions where nucleation thresholds can be reached even when domain-median conditions would not favor nucleation. This highlights the importance of resolving spatial variability when interpreting the timing and variability of new particle formation.

S6.4 Grid-resolution sensitivity analysis

To assess the sensitivity of the simulated flow and NPF coupling to spatial resolution, we performed a grid-resolution analysis using grid spacings of 2000, 1000, 500, 300, 200, 100, 50, 20, 10, and 5 m under identical weak-wind forcing conditions.

Figure S6.6 shows the variation of turbulent kinetic energy (TKE) with grid resolution, while Fig. S6.7 presents the corresponding turbulence length scale (L_t). At very coarse resolutions (2000–1000 m), TKE is low and L_t is unrealistically large, indicating that the dominant flow structures are not resolved. At intermediate resolutions (500–300 m), TKE exhibits non-monotonic behavior, suggesting sensitivity to numerical resolution in this transition regime.

At finer resolutions ($\Delta x \lesssim$ 20–30 m), both TKE and L_t become much less sensitive to further refinement, indicating convergence of the dominant flow characteristics. In particular, the differences between the 10 m and 5 m simulations are small compared with those observed at coarser resolutions.

These results indicate that a grid spacing of order 10 m is sufficient to capture the key flow–transport structures relevant for NPF under the conditions considered here. While further refinement may influence local peak values and the sharpness of gradients, it does not materially alter the overall spatial patterns or the process-level coupling between flow, vapor transport, and particle formation.

Together with the spatial correspondence between H_2SO_4, nucleation rate, and particle fields, this analysis supports the interpretation that the observed heterogeneity in NPF is physically driven rather than a numerical artefact.

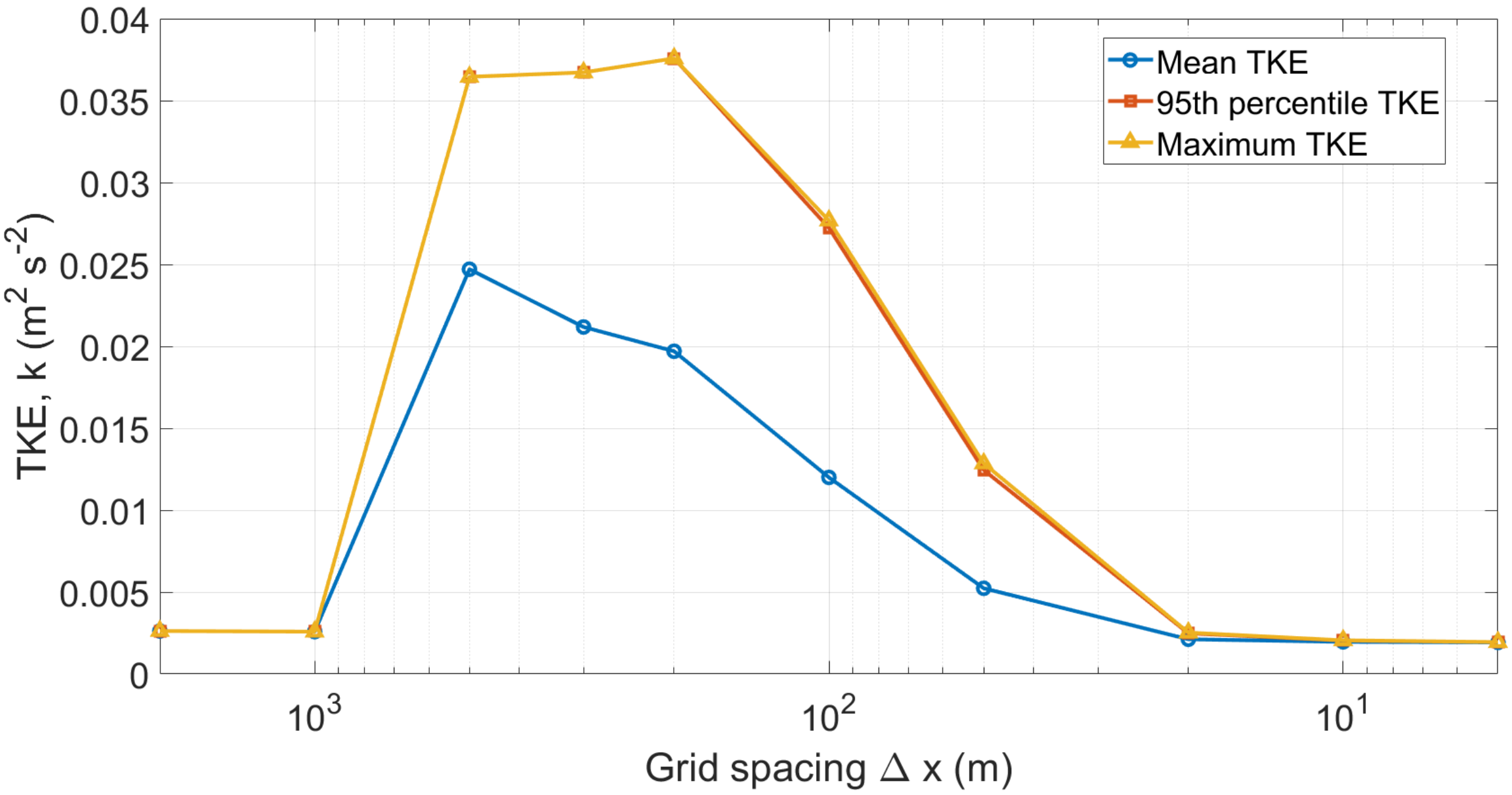

Figure S6.6: Dependence of turbulent kinetic energy (TKE, $\mathrm{m^2\,s^{-2}}$) on grid spacing (Δx). Shown are the domain-averaged mean, 95th percentile, and maximum TKE across simulations with progressively refined resolution. TKE increases as the grid is refined from kilometre to hectometre scales, reflecting improved resolution of turbulent motions, and reaches a plateau for$\Delta x < 20\,\mathrm{m}$, indicating convergence of resolved turbulence.

S6.5 Turbulence length scale and resolution requirements

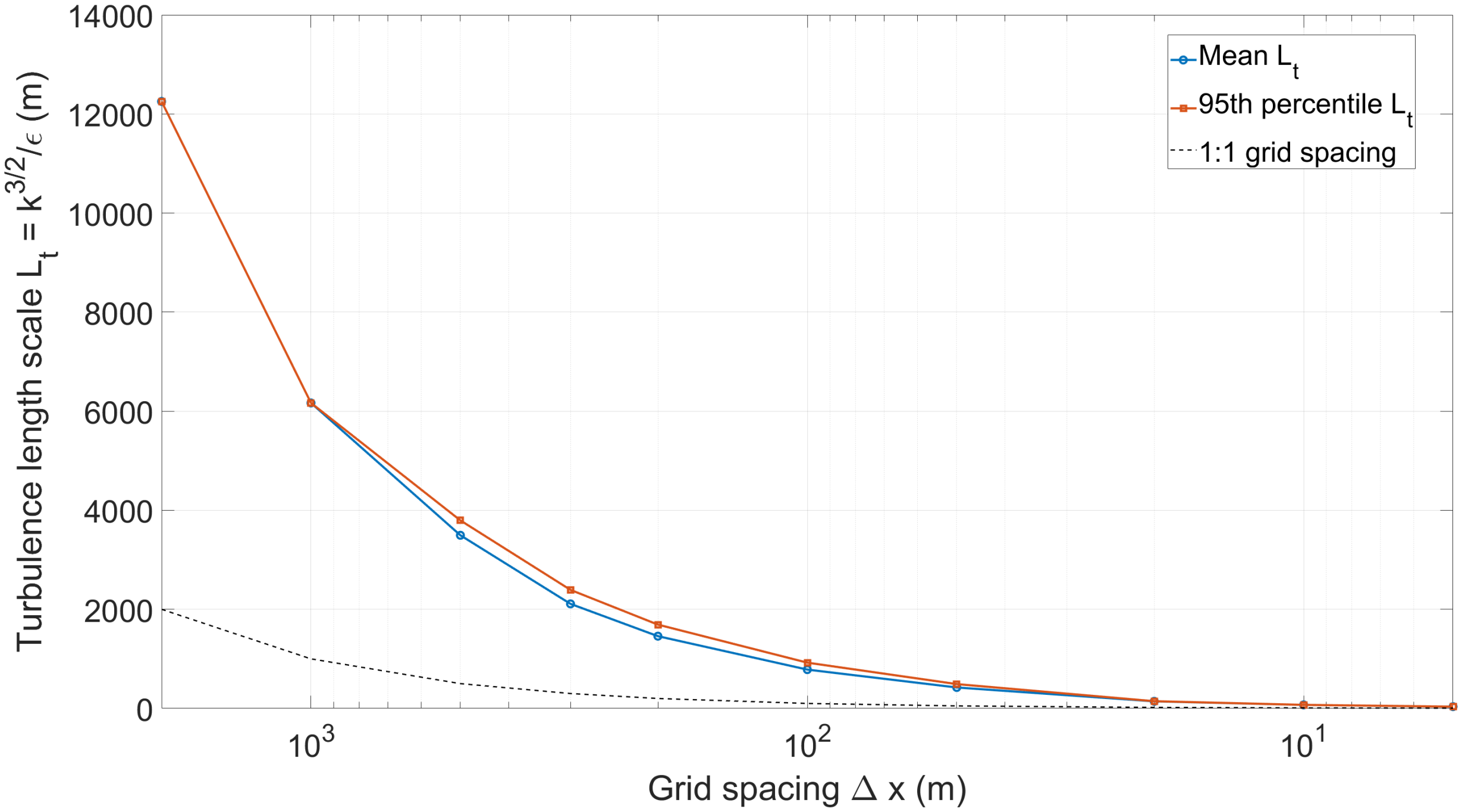

Figure S6.7: Turbulence length scale ($L_t = k^{3/2}/\epsilon$, m) as a function of grid spacing (Δx). The mean and 95th percentile values are shown. The dashed line indicates the 1:1 relationship between L_t and grid spacing. At coarse resolution, $L_t \gg \Delta x$, indicating under-resolved turbulence. As resolution increases, L_t approaches Δx, suggesting that the dominant turbulent eddies become increasingly resolved.

S6.6 Banana plots with 'source-only' nucleation

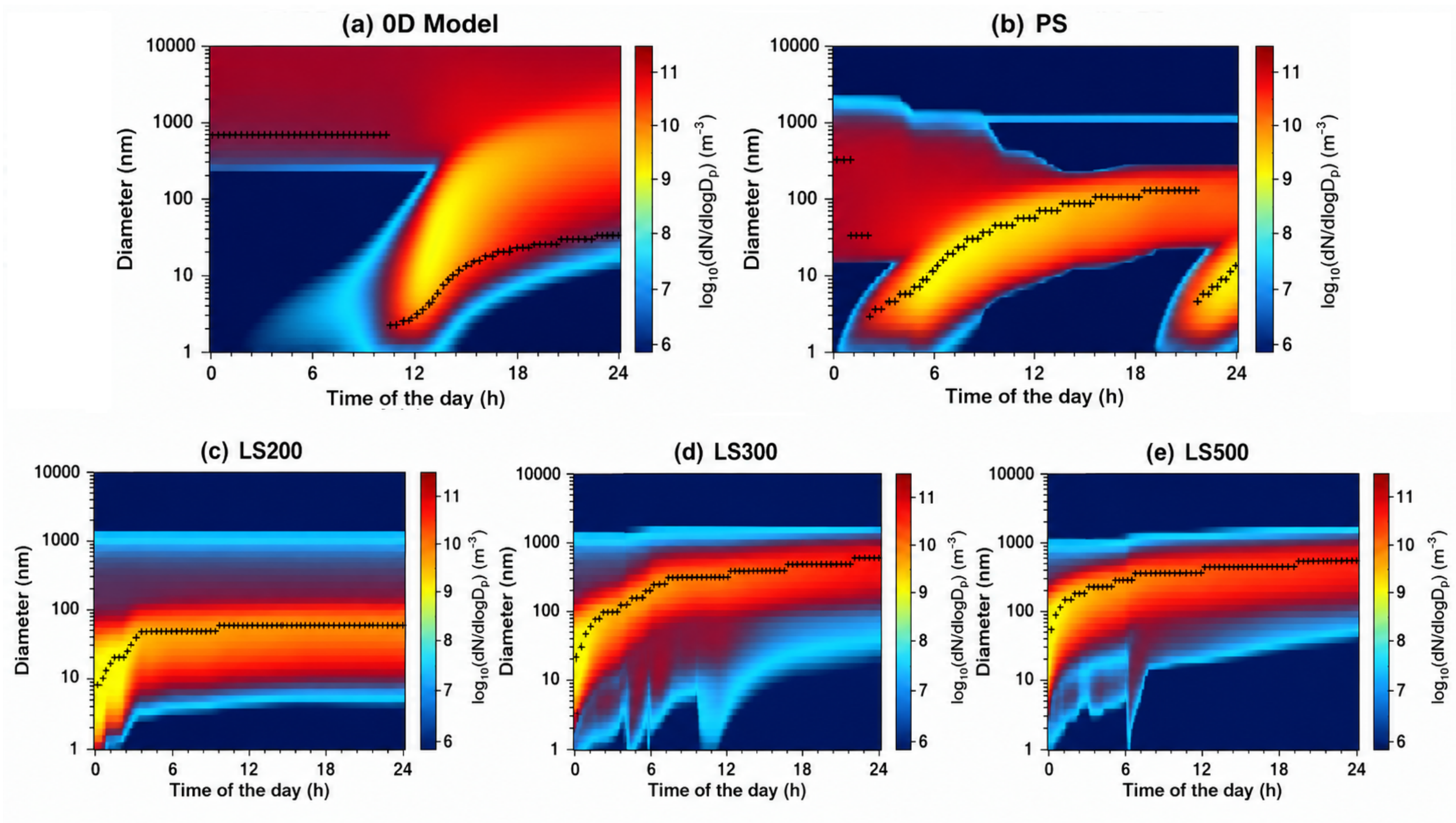

Figure S6.8: Time evolution of particle number size distributions ($\log_{10}(dN/d\log D_p)$) at the central grid point (30 m height) for the 0D reference model (panel a) and the four 3D emission cases (panels b–e). Color contours represent the logarithmic particle number concentration. The black line indicates the geometric mean diameter of the fitted nucleation mode (NPF mode) over time, obtained from mode fitting of the particle number size distributions.

S6.7 Growth rates

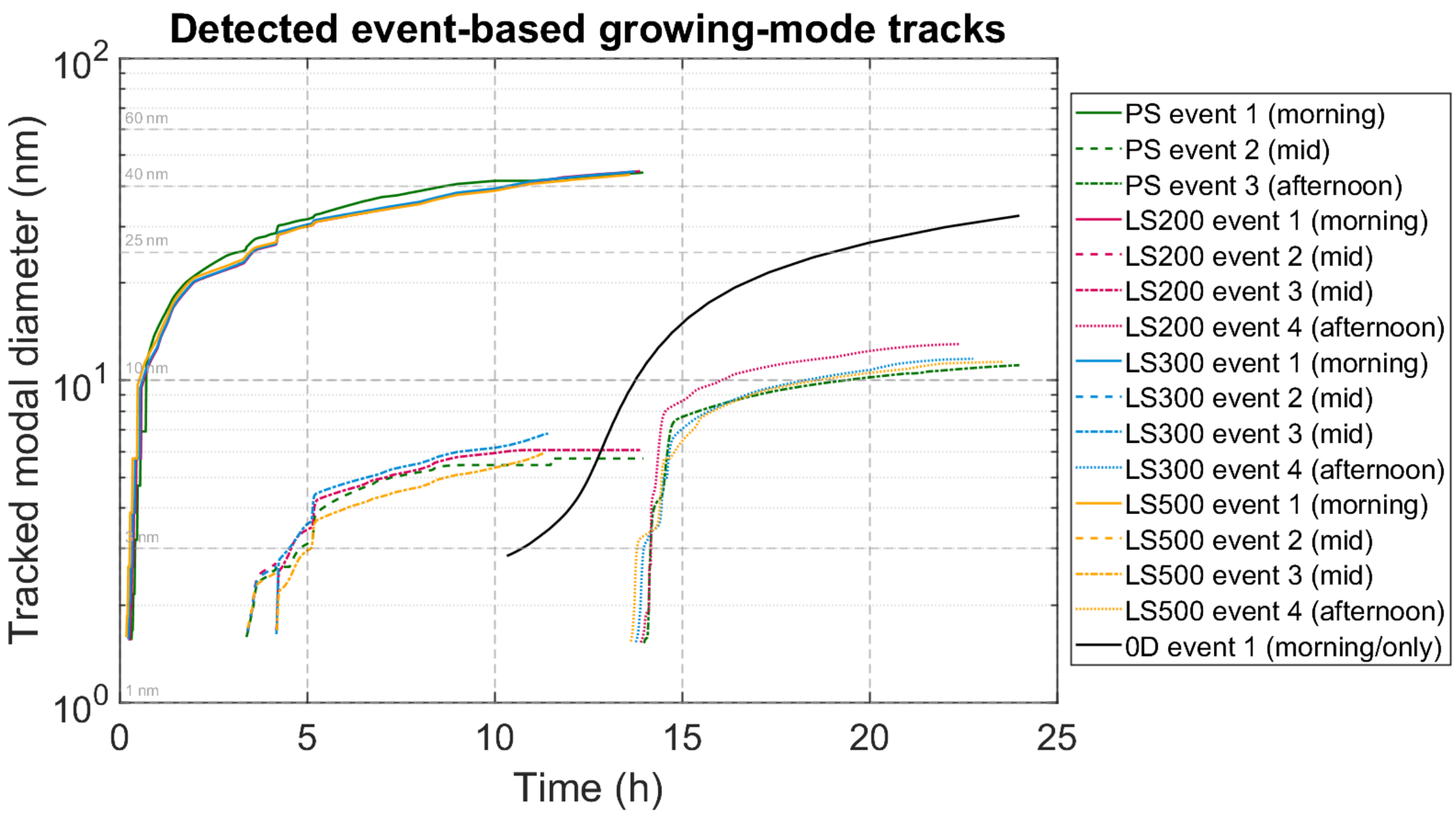

Figure S6.9: Detected event-based growing mode tracks over the 24-hr simulation time for all 3D and the 0D scenarios contributing to calculation of growth rates in Figure 8 of the main text.

Table S6.1: Event-based median growth rate (GR) in nm h^{-1}.

Bin	PS	LS200	LS300	LS500	0D
1–3	8.307	5.956	6.827	8.772	0.387
3–10	13.842	13.618	12.499	13.350	2.309
10–25	5.974	5.135	5.121	5.093	2.839
25–40	2.739	2.153	2.215	2.093	1.493
40–60	0.785	1.378	1.308	1.186	–

Table S6.2: Event-based robust spread in GR, in nm h^{-1}.

Bin	PS	LS200	LS300	LS500	0D
1–3	10.858	3.796	4.008	10.820	–
3–10	18.534	14.759	16.125	17.644	–
10–25	–	–	–	–	–
25–40	–	–	–	–	–
40–60	–	–	–	–	–

Table S6.3: Number of event GR values used after full-crossing preference.

Bin	PS	LS200	LS300	LS500	0D
1–3	3	3	3	3	1
3–10	2	2	2	2	1
10–25	1	1	1	1	1
25–40	1	1	1	1	1
40–60	1	1	1	1	–

Table S6.4: Percentage change in GR for each case relative to 0D median GR. The formula used is $100 \times (GR_{case} - GR_{0D})/GR_{0D}$.

Region	PS (%)	LS200 (%)	LS300 (%)	LS500 (%)	RMSE (nm h^{-1})
1–3 nm	2047.65	1439.89	1665.01	2167.83	7.1684
a (3–10 nm)	499.46	489.77	441.32	478.17	11.0302
b (10–25 nm)	110.44	80.86	80.37	79.40	2.5194
c (25–40 nm)	83.52	44.26	48.37	40.24	0.8474

Table S6.5: Size-resolved observational nanoparticle growth rates (GR) mapped to the model size bins. Only studies reporting explicit size intervals, or intervals that map cleanly onto the model bins, are included.

**Study**	**Site / type**	**Size range (nm)**	**Reported GR (nm h^{-1})**
**1–3 nm**			
Yli-Juuti et al. (2011)[S10]	Hyytiälä, boreal/rural	1.5–3	1.9
Baalbaki et al. (2021)[S11]	Agia Marina, Cyprus/rural	1.5–3	3.7
Yu et al. (2016)[S12]	Nanjing, urban polluted	1.4–3	4.3
Deng & Cai et al. (2021)[S13]	Beijing, urban polluted	1.5–3	2.6
Xiao et al. (2015)[S14]	Shanghai, urban polluted	1.35–2.39	0.94
**3–10 nm**			
Yli-Juuti et al. (2011)[S10]	Hyytiälä, boreal/rural	3–7	3.8
Baalbaki et al. (2021)[S11]	Agia Marina, Cyprus/rural	3–7	9.2
Yao et al. (2018)[S15]	Shanghai, urban polluted	3–7	6.5
Xiao et al. (2015)[S14]	Shanghai, urban polluted	2.39–7	10.9

continued on next page

Table S6.5 – *continued from previous page*

**Study**	**Site / type**	**Size range (nm)**	**Reported GR ($nm\,h^{-1}$)**
Manninen et al. (2010)[S16]	Pallas, remote	3–7	5.6
Manninen et al. (2010)[S16]	Mace Head, remote/coastal	3–7	2.7
Manninen et al. (2010)[S16]	Cabauw, rural	3–7	5.5
Manninen et al. (2010)[S16]	Melpitz, rural	3–7	5.7
Manninen et al. (2010)[S16]	Hohenpeissenberg, rural	3–7	4.2
Manninen et al. (2010)[S16]	Jungfraujoch, high altitude	3–7	7.2
Kontkanen et al. (2016)[S17]	Po Valley, rural/polluted	3–7	6.0
Deng et al. (2020)[S18]	Beijing winter, urban	3–7	2.4
Deng et al. (2020)[S18]	Beijing spring, urban	3–7	2.4
Deng et al. (2020)[S18]	Beijing summer, urban	3–7	2.8
Deng et al. (2020)[S18]	Beijing autumn, urban	3–7	1.7
Jokinen et al. (2018)[S19]	Aboa, Antarctica/remote	6–10	0.49
Brean et al. (2020)[S20]	Barcelona, urban	1.9–4.5	4.4
Brilke et al. (2020)[S21]	Paphos, Cyprus	3.2–10	5.5
Jayaratne et al. (2017)[S22]	Beijing, urban	2–10	3.5
Dai et al. (2017)[S23]	Yangtze R. Delta, urban/regional	3–10	6.5
Brean et al. (2021)[S24]	Antarctic Peninsula/remote	4.5–10	0.5
**10–25 nm**			
Yli-Juuti et al. (2011)[S10]	Hyytiälä, boreal/rural	7–20	4.3
Baalbaki et al. (2021)[S11]	Agia Marina, Cyprus/rural	7–20	11.7
Yao et al. (2018)[S15]	Shanghai, urban polluted	7–25	9.9
Xiao et al. (2015)[S14]	Shanghai, urban polluted	7–20	11.4
Manninen et al. (2010)[S16]	Pallas, remote	7–20	3.8
Manninen et al. (2010)[S16]	Mace Head, remote/coastal	7–20	5.4
Manninen et al. (2010)[S16]	Cabauw, rural	7–20	6.7
Manninen et al. (2010)[S16]	Melpitz, rural	7–20	7.0
Manninen et al. (2010)[S16]	Hohenpeissenberg, rural	7–20	4.6
Manninen et al. (2010)[S16]	Jungfraujoch, high altitude	7–20	8.2

continued on next page

Table S6.5 – *continued from previous page*

**Study**	**Site / type**	**Size range (nm)**	**Reported GR ($nm\,h^{-1}$)**
Kontkanen et al. (2016)[S17]	Po Valley, rural/polluted	7–15	7.2
Deng et al. (2020)[S18]	Beijing winter, urban	7–15	3.0
Deng et al. (2020)[S18]	Beijing spring, urban	7–15	3.4
Deng et al. (2020)[S18]	Beijing summer, urban	7–15	4.0
Deng et al. (2020)[S18]	Beijing autumn, urban	7–15	3.6
Brean et al. (2020)[S20]	Barcelona, urban	4.5–20	4.7
Boulon et al. (2010)[S25]	Jungfraujoch, high altitude	7–20	5.7
Rose et al. (2015)[S26]	Chacaltaya, high altitude	7–20	7.6
**25–40 nm**			
Mishra et al. (2023)[S27]	Delhi, urban polluted	20–40	22.3 ± 19.4
Hao et al. (2025)[S28]	Chongqing, urban	25–40	7.12 ± 2.05
Hao et al. (2025)[S28]	Chongqing, urban	25–40	9.22 ± 4.28

References

(S1) Ghosh, K.; Tripathi, S. N.; Joshi, M.; Mayya, Y. S.; Khan, A.; Sapra, B. K. Particle formation from vapors emitted from glowing wires: Theory and experiments. *Aerosol Science and Technology* **2020**, *54*, 243–261.

(S2) Brean, J.; Rowell, A.; Beddows, D. C. S.; Shi, Z.; Harrison, R. M. Estimates of Future New Particle Formation under Different Emission Scenarios in Beijing. *Environmental Science & Technology* **2023**, *57*, 4741–4750, PMID: 36930743.

(S3) Uhrner, U.; von Löwis, S.; Vehkamäki, H.; Wehner, B.; Bräsel, S.; Hermann, M.; Stratmann, F.; Kulmala, M.; Wiedensohler, A. Dilution and aerosol dynamics within a diesel car exhaust plume—CFD simulations of on-road measurement conditions. *Atmospheric Environment* **2007**, *41*, 7440–7461.

(S4) Frederick, S. G.; Mohebalhojeh, M.; Curtis, J. H.; West, M.; Riemer, N. Idealized particle-resolved large-eddy simulations to evaluate the impact of emissions spatial heterogeneity on CCN activity. *Atmospheric Chemistry and Physics* **2026**, *26*, 4863–4883.

(S5) Vohra, K.; Ghosh, K.; Tripathi, S.; Thangamani, I.; Goyal, P.; Dutta, A.; Verma, V. Submicron particle dynamics for different surfaces under quiescent and turbulent conditions. *Atmospheric Environment* **2017**, *152*, 330–344.

(S6) Ghosh, K.; Tripathi, S.; Joshi, M.; Mayya, Y.; Khan, A.; Sapra, B. Modeling studies on coagulation of charged particles and comparison with experiments. *Journal of Aerosol Science* **2017**, *105*, 35–47.

(S7) Mishra, S.; Tripathi, S. N.; Kanawade, V. P.; Haslett, S. L.; Dada, L.; Ciarelli, G.; Kumar, V.; Singh, A.; Bhattu, D.; Rastogi, N.; Daellenbach, K. R.; Ganguly, D.; Gargava, P.; Slowik, J. G.; Kulmala, M.; Mohr, C.; El-Haddad, I.; Prevot, A. S. H. Rapid night-time nanoparticle growth in Delhi driven by biomass-burning emissions. *Nature Geoscience* **2023**, *16*, 224–230.

(S8) Cai, R.; Yan, C.; Worsnop, D. R.; Bianchi, F.; Kerminen, V.-M.; Liu, Y.; Wang, L.; Zheng, J.; Kulmala, M.; Jiang, J. An indicator for sulfuric acid–amine nucleation in atmospheric environments. *Aerosol Science and Technology* **2021**, *55*, 1059–1069.

(S9) Stolzenburg, D.; Sarnela, N.; Bianchi, F.; Cai, J.; Cai, R.; Cheng, Y.; Dada, L.; Donahue, N. M.; Grothe, H.; Holm, S.; Kerminen, V.-M.; Lehtipalo, K.; Petäjä, T.; Sulo, J.; Winkler, P. M.; Yan, C.; Kangasluoma, J.; Kulmala, M. Incomplete mass closure in atmospheric nanoparticle growth. *npj Climate and Atmospheric Science* **2025**, *8*, 75.

(S10) Yli-Juuti, T.; Nieminen, T.; Hirsikko, A.; Aalto, P. P.; Asmi, E.; Hõrrak, U.; Manninen, H. E.; Patokoski, J.; Dal Maso, M.; Petäjä, T.; Rinne, J.; Kulmala, M.; Riipinen, I. Growth rates of nucleation mode particles in Hyytiälä during 2003–2009: variation with particle size, season, data analysis method and ambient conditions. *Atmospheric Chemistry and Physics* **2011**, *11*, 12865–12886.

(S11) Baalbaki, R.; Pikridas, M.; Jokinen, T.; Laurila, T.; Dada, L.; Bezantakos, S.; Ahonen, L.; Neitola, K.; Maisser, A.; Bimenyimana, E.; Christodoulou, A.; Unga, F.; Savvides, C.; Lehtipalo, K.; Kangasluoma, J.; Biskos, G.; Petäjä, T.; Kerminen, V.-M.; Sciare, J.; Kulmala, M. Towards understanding the characteristics of new particle formation in the Eastern Mediterranean. *Atmospheric Chemistry and Physics* **2021**, *21*, 9223–9251.

(S12) Yu, H.; Zhou, L.; Dai, L.; Shen, W.; Dai, W.; Zheng, J.; Ma, Y.; Chen, M. Nucleation and growth of sub-3 nm particles in the polluted urban atmosphere of a megacity in China. *Atmospheric Chemistry and Physics* **2016**, *16*, 2641–2657.

(S13) Deng, C.; Cai, R.; Yan, C. Formation and growth of sub-3 nm particles in megacities: impact of background aerosols. *Faraday Discussions* **2021**, *226*, 348–363.

(S14) Xiao, S.; Wang, M. Y.; Yao, L.; Kulmala, M.; Zhou, B.; Yang, X.; Chen, J. M.; Wang, D. F.; Fu, Q. Y.; Worsnop, D. R.; Wang, L. Strong atmospheric new particle formation in winter in urban Shanghai, China. *Atmospheric Chemistry and Physics* **2015**, *15*, 1769–1781.

(S15) Yao, L.; Garmash, O.; Bianchi, F.; Zheng, J.; Yan, C.; Kontkanen, J.; Junninen, H.; Mazon, S. B.; Ehn, M.; Paasonen, P.; SipilÃ¤, M.; Wang, M.; Wang, X.; Xiao, S.; Chen, H.; Lu, Y.; Zhang, B.; Wang, D.; Fu, Q.; Geng, F.; Li, L.; Wang, H.; Qiao, L.; Yang, X.; Chen, J.; Kerminen, V.-M.; PetÃ¤jÃ¤, T.; Worsnop, D. R.; Kulmala, M.; Wang, L. Atmospheric new particle formation from sulfuric acid and amines in a Chinese megacity. *Science* **2018**, *361*, 278–281.

(S16) Manninen, H. E.; Nieminen, T.; Asmi, E.; Gagné, S.; Häkkinen, S.; Lehtipalo, K.; Aalto, P.; Vana, M.; Mirme, A.; Mirme, S.; Hõrrak, U.; Plass-Dülmer, C.; Stange, G.; Kiss, G.; Hoffer, A.; Törö, N.; Moerman, M.; Henzing, B.; de Leeuw, G.; others EUCAARI ion spectrometer measurements at 12 European sites – analysis of new particle formation events. *Atmospheric Chemistry and Physics* **2010**, *10*, 7907–7927.

(S17) Kontkanen, J.; Järvinen, E.; Manninen, H. E.; Lehtipalo, K.; Kangasluoma, J.; Decesari, S.; Gobbi, G. P.; Laaksonen, A.; Petäjä, T.; Kulmala, M. High concentrations of sub-3 nm clusters and frequent new particle formation observed in the Po Valley, Italy, during the PEGASOS 2012 campaign. *Atmospheric Chemistry and Physics* **2016**, *16*, 1919–1935.

(S18) Deng, C.; Cai, R.; Yan, C.; others Seasonal characteristics of new particle formation and growth in urban Beijing. *Environmental Science & Technology* **2020**, *54*, 8547–8557.

(S19) Jokinen, T.; Sipilä, M.; Junninen, H.; Ehn, M.; Lönn, G.; Hakala, J.; Petäjä, T.; Mauldin, R. L.; Kulmala, M.; Worsnop, D. R.; Kirkby, J.; Virkkula, A.; Kerminen, V.-M.; others Ion-induced sulfuric acid–ammonia nucleation drives particle formation in coastal Antarctica. *Science Advances* **2018**, *4*, eaat9744.

(S20) Brean, J.; Beddows, D. C. S.; Shi, Z.; Temime-Roussel, B.; Marchand, N.; Querol, X.; Alastuey, A.; Minguillón, M. C.; Harrison, R. M. Molecular insights into new particle formation in Barcelona, Spain. *Atmospheric Chemistry and Physics* **2020**, *20*, 10029–10045.

(S21) Brilke, S.; Fölker, N.; Müller, T.; Kandler, K.; Gong, X.; Peischl, J.; Weinzierl, B.; Winkler, P. M. New particle formation and sub-10 nm size distribution measurements during the A-LIFE field experiment in Paphos, Cyprus. *Atmospheric Chemistry and Physics* **2020**, *20*, 5645–5656.

(S22) Jayaratne, R.; Pushpawela, B.; He, C.; Li, H.; Gao, J.; Chai, F.; Morawska, L. Observations of particles at their formation sizes in Beijing, China. *Atmospheric Chemistry and Physics* **2017**, *17*, 8825–8835.

(S23) Dai, L.; Wang, H. L.; Zhou, L. Y.; An, J. L.; Tang, L. L.; Lu, C. S.; Yan, W. L.; Liu, R. Y.; Kong, S. F.; Chen, M. D.; Lee, S. H.; Yu, H. Regional and local new particle formation events observed in the Yangtze River Delta region, China. *Journal of Geophysical Research: Atmospheres* **2017**, *122*, 2389–2402.

(S24) Brean, J.; Dall'Osto, M.; Simó, R.; Shi, Z.; Beddows, D. C. S.; Harrison, R. M. Open ocean and coastal new particle formation from sulfuric acid and amines around the Antarctic Peninsula. *Nature Geoscience* **2021**, *14*, 383–388.

(S25) Boulon, J.; Sellegri, K.; Venzac, H.; Picard, D.; Weingartner, E.; Wehrle, G.; Collaud Coen, M.; Bütikofer, R.; Flückiger, E.; Baltensperger, U.; Laj, P. New particle formation and ultrafine charged aerosol climatology at a high altitude site in the Alps (Jungfraujoch, 3580 m a.s.l., Switzerland). *Atmospheric Chemistry and Physics* **2010**, *10*, 9333–9349.

(S26) Rose, C.; Sellegri, K.; Velarde, F.; Moreno, I.; Ramonet, M.; Weinhold, K.; Krejci, R.; Andrade, M.; Wiedensohler, A.; Laj, P. Frequent nucleation events at the high altitude station of Chacaltaya (5240 m a.s.l.), Bolivia. *Atmospheric Environment* **2015**, *102*, 18–29.

(S27) Mishra, S.; Tripathi, S. N.; Kanawade, V. P.; Haslett, S. L.; Dada, L.; Ciarelli, G.; Kumar, V.; Singh, A.; Bhattu, D.; Rastogi, N.; Daellenbach, K. R.; Ganguly, D.; Gargava, P.; Slowik, J. G.; Kulmala, M.; Mohr, C.; El-Haddad, I.; Prévôt, A. S. H. Rapid night-time nanoparticle growth in Delhi driven by biomass-burning emissions. *Nature Geoscience* **2023**, *16*, 224–230.

(S28) Hao, Y.; Li, P.; Gou, Y.; Wang, Z.; Tian, M.; Chen, Y.; Kuang, Y.; others Divergent changes in aerosol optical hygroscopicity and new particle formation during a heatwave of summer 2022. *Atmospheric Chemistry and Physics* **2025**, *25*, 12811–12830.
